# Synergistic Cosensitization and Redox-Triggered Interfacial Engineering for Efficient and Durable Solar Cells

**DOI:** 10.1021/acsami.6c01394

**Published:** 2026-04-28

**Authors:** Heng Wu, Laia Marín Moncusí, Javier Perez Hernandez, Eugenia Martinez-Ferrero, Emilio Palomares

**Affiliations:** † 202569 Institute of Chemical Research of Catalonia (ICIQ)-CERCA, Avinguda Països Catalans, 16, Tarragona 43007, Spain; ‡ Universitat Rovira i Virgili (URV), Departament D’enginyeria electrònica Elèctrica i Automàtica, Avinguda Països Catalans, 26, Tarragona 43007, Spain; § Catalan Institution for Research and Advanced Studies (ICREA), Passeig Lluïs Companys, 23, Barcelona 08010, Spain; ∥ School of Chemistry and Chemical Engineering, Key Laboratory of Electrochemical Energy Storage and Energy Conversion of Hainan Province, Key Laboratory of Electrochemical Energy Storage and Light Energy Conversion Materials of Haikou City, Hainan Normal University, Haikou 571158, China

**Keywords:** dye sensitized solar cells, interfacial engineering, cosensitization, excited state, charge transfer, hypervalent iodine, long-term stability

## Abstract

Achieving long-term stability along with high power conversion efficiency (PCE) remains a critical challenge for dye-sensitized solar cells (DSCs). Herein, we report a synergistic strategy that combines cosensitization and redox-active interfacial engineering to enhance the performance and durability of DSC. A narrow-energy-gap sensitizer (**H4**) is paired with a complementary blue-light-absorbing dye (**H15**), which possesses a strong absorption at ∼410 nm and a prolonged excited-state lifetime, thereby compensating for the spectral response and reducing interfacial charge recombination in cografted titania films. Simultaneously, we introduce a hypervalent iodine­(III) compound, 1-acetoxy-1,2-benziodoxol-3­(1*H*)-one (IBA), into a cobalt-based electrolyte. Initially, the introduction of IBA facilitates fast oxidation of the triphenylamine electron donor in **H4**, generating free radicals and enhancing intramolecular charge transfer. Importantly, the redox byproduct 2-iodobenzoic acid (IA) plays a critical role in suppressing interfacial recombination and passivation by coordinating with lithium ion and forming halogen-bonded complexes with electrolyte additives. The synergistic effects of cosensitization and the IBA additive in the electrolyte yield a cosensitized DSC with a PCE of 12.84%, featuring excellent operational stability under indoor light soaking for 1000 h. Moreover, the control device achieves an efficiency of 25.81% when tested under indoor light (4500 lx) illumination.

## Graphical abstract

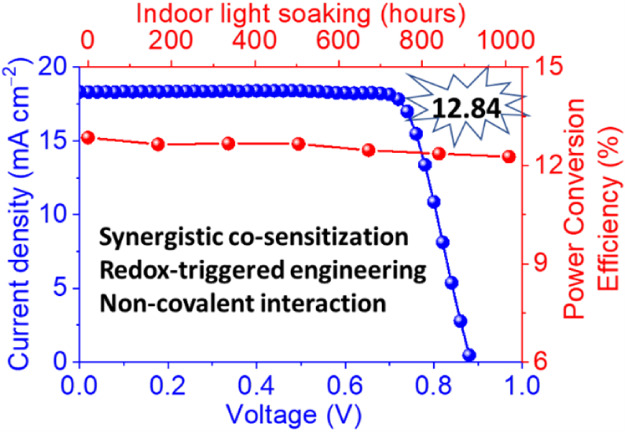

## 1 Introduction

Dye-sensitized solar cells (DSCs), as a low-cost photovoltaic technology, have garnered significant academic and commercial attention since the pioneering work of O’Regan and Grätzel.^1^ The performance of DSCs is primarily driven by enhanced incident photon-to-electron conversion efficiency (IPCE)^2,3^ and increased open-circuit photovoltage (*V*_oc_), enabled through strategic molecular design,^4,5^ careful electrolyte formulation,^6–10^ and interfacial engineering,^11–13^ which together influence the excited-state dynamics of the photosensitizer and mitigate interfacial charge recombination. Achieving excellent long-term operational stability remains a prerequisite for industrial deployment. Historically, DSCs employing iodide/triiodide redox couples in ionic liquid electrolytes have led the field, offering substantial durability under continuous full-sun illumination.^14,15^ Nowadays, the strategy to fabricate high-efficiency DSCs is based on the synergistic effect of multiple dyes cosenstization to achieve spectral complementation and interface optimization.^16–21^ For instance, Wang and coworkers developed a coadsorption strategy involving the narrow-energy-gap C268 and wide-bandgap SC-4, forming a robust self-assembled dye molecule monolayer that enabled a stable ionic liquid-based DSC with a power conversion efficiency (PCE) of 10%.^17^ Meanwhile, Hanaya and coworkers cografted two organic dyes, ADEKA-1 and LEG4, onto the surface of titanium dioxide. Subsequently, the pinhole defects within the dye layer were repaired through nanoscale multilevel passivation, enabling a power conversion efficiency as high as 14.5%.^18^ On the other hand, the record PCE of DSC is held by Ren et al. that reported a preadsorption method to form a densely packed, ordered layer of dye molecules on mesoporous titania (TiO_2_), yielding a record PCE of 15.2%.^21^ Although copper-based redox mediators provide higher redox potentials and consequently enable improved open-circuit voltages compared to those of the conventional iodide/triiodide couple, their application in DSCs is often accompanied by reduced photocurrent densities. This limitation arises from several factors, including the decreased thermodynamic driving force for dye regeneration, the slow diffusion of bulky Cu­(II) species, increased interfacial charge recombination, and kinetic limitations associated with structural reorganization during the Cu­(I)/Cu­(II) redox cycle.

In recent years, cobalt-based electrolytes have revitalized the DSC field due to their higher open-circuit voltages, low corrosivity toward metals, and excellent visible-light transparency.^10,18,22^ Furthermore, they have been widely investigated for indoor DSCs owing to their high open-circuit voltage and low charge recombination, and displaying impressive PCEs of over 25% under indoor lighting, superior to other thin-film semiconductor photovoltaic technologies.^23,24^ Rational ligand design and engineering enable fine control over the electrochemical properties of cobalt-based coordination complexes.^25^ Nevertheless, this system requires the use of electrolyte additives such as lithium bis­(trifluoromethanesulfonyl)­imide (LiTFSI) and *N*-methylimidazole (NMB) or 4-*tert*-butylpyridine (TBP) that play critical roles in optimizing the performance of DSCs, yet both exhibit notable adverse effects. Li^+^ ions can strongly adsorb on the TiO_2_ surface, which causes a downward shift of the TiO_2_ conduction band edge that leads to a reduction of the photovoltage.^26^ Similarly, NMB molecules tend to adsorb on the TiO_2_ surface and compete with the dye molecules for active adsorption sites, which can lead to partial dye desorption and a consequent decrease in light-harvesting capability.^27^ Additionally, LiTFSI and NMB increase the viscosity of the electrolyte, which hinders the diffusion of the cobalt­(II)/cobalt­(III) redox couple, leading to higher mass transport resistance and potentially lowering the fill factor.^28^ Furthermore, at high concentrations or under prolonged operation, the presence of Li^+^ can also accelerate electrode corrosion and negatively impact device stability.^29^ Long-term stability in cobalt-based systems remains underexplored, largely due to challenges such as dye desorption, solvent evaporation, and instability of the cobalt redox mediator—arising from ligand dissociation and deleterious cation effects.^30–32^ Recent electrolyte innovations,^33^ including the removal of Li^+^ additives^34^ and the use of polydentate ligands,^35^ have shown promise in improving stability. Yet, achieving both high efficiency and robust long-term performance remains a challenge for cobalt-based DSCs, particularly when compared to iodide/triiodide systems.

To address this, we have introduced a novel additive: a hypervalent iodine­(III) compound, 1-acetoxy-1,2-benziodoxol-3­(1*H*)-one (IBA) (Figure S1) into cobalt-based electrolytes.^36,37^ IBA is a bench-stable organic oxidant widely used in organic synthesis for mild, selective oxidations, including the transformation of alcohols and amines as well as C–H functionalization reactions. Its unique reactivity arises from its cyclic iodine­(III) structure, which facilitates efficient single- or two-electron-transfer processes with good functional group tolerance. During redox activity, IBA converts to 2-iodobenzoic acid (IA), a benign byproduct (Figure S1). By leveraging IBA’s coordination ability and redox stability, we aim to suppress Li^+^ and NMB accumulation at the TiO_2_ interface and improve both the efficiency and long-term operational stability of cobalt-based DSCs.

In this work, we develop a narrow-energy-gap sensitizer **H4** that exhibits a broad spectral response across the entire visible light region. We reasoned that to complement the light-harvesting capability of **H4**, a cosensitizer exhibiting strong absorption at around 400 nm was necessary. Ideally, this cosensitizer, together with better passivation of the TiO_2_ surface, would reduce interfacial charge recombination, leading to a solar cell with a higher open-circuit voltage (*V*_oc_) and short-circuit photocurrent (*J*_sc_). To achieve these goals, we conceived and synthesized **H15**, incorporating a bulky triarylamine donor and a 2-ethylhexyl-substituted benzotriazole unit (Figure 1a). The presence of the alkyl substituents improves the solubility and reduces interfacial charge recombination. **H15** exhibits a strong absorption peak around 410 nm with a high molar extinction coefficient, effectively compensating for the inefficient blue light harvesting of **H4**.

**Figure 1:**
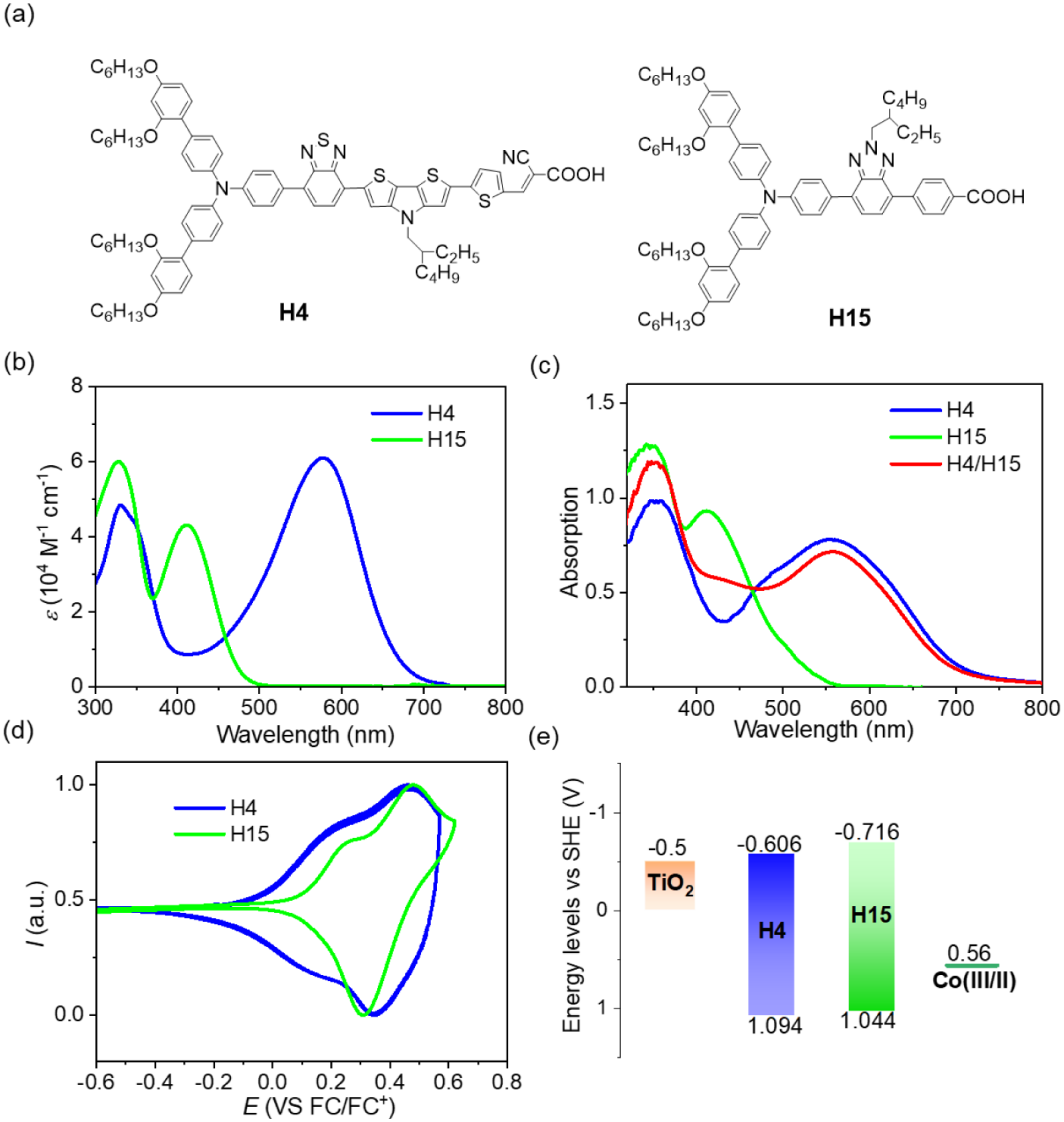
(a) Molecular structure of dyes **H4** and **H15**. (b) UV–vis absorption spectra of the dyes dissolved in dichloromethane (0.1 M). (c) UV–vis absorption spectra of **H4**, **H15**, and **H4**/**H15** cosensitized 2.0-μm-thick transparent nanocrystalline mesoporous titanium dioxide (TiO_2_) films after 12 h 0.1 M dye solution soaking. (d) Cyclic voltammogram curves of dyes adsorbed on 4 μm thick mesoscopic TiO_2_ films supported by FTO electrodes in acetonitrile using a 0.1 M tetrabutylammonium hexafluorophosphate (TBAPF_6_) solution as the supporting electrolyte. The scan rate is 50 mV s^–1^, and Fc/Fc^+^ is the external reference. (e) Energy levels diagram of TiO_2_, dyes, and [Co­(II)­(bpy)_3_]­[TFSI]_2_ vs SHE.

Initially, the IBA possesses a suitable oxidation capacity, which can capture electrons from triphenylamine (TPA) in the **H4** molecule through an oxidation–reduction reaction. This instant reaction can generate TPA•^+^ species and 2-iodobenzoic acid. Figure S1 provides a comprehensive schematic diagram illustrating the multistep mechanism of IBA. By gas chromatography (GC) we have confirmed the detection of the byproduct IA (Figure S2). Furthermore, the formation of the radical cation TPA•^+^ has been evaluated by electron paramagnetic resonance (EPR) experiments in chloroform solutions at room temperature. No radical signals are observed for the dye solution in chloroform and the IBA solution (10^–4^ M). However, when the dye solution was mixed with IBA, the EPR spectrum showed three main peaks (Figure S3). This hyperfine pattern can be assigned to the radical located on the nitrogen atom of TPA.^38,39^ Interestingly, the formation of the radical cation TPA•^+^ enhances the intramolecular charge transfer (ICT) and hole injection.^40,41^

Importantly, the fast oxidation process demonstrated that the introduction of IBA facilitates hole injection from oxidized dye molecules to Cobalt­(II). The generated IA, a molecule possessing a halogen bond donor,^42–44^ can serve as a locker of *N*-methylimidazole (NMB) by forming an NMB-DB complex^45–48^ through the chemical binding of a halogen bond (I···N), which can effectively suppress the ion mobility and prevent ion aggregation toward the titania surface. Also, the coordination between the carbonyl oxygen and Li^+^ ions (COO^–^···Li^+^) can hinder Li^+^ migration toward the titania surface, thereby suppressing interfacial charge recombination.^49,50^ Those multi-interactions based on the introduction of IBA and IA-mediated interfacial passivation boost the cosensitized solar cell to exhibit a power conversion efficiency of up to 12.84% with good reproducibility and long-term operational stability. Furthermore, the control device displays an outstanding efficiency of up to 25.81% under an indoor light.

## 2 Results and Discussion

### 2.1 Syntheses of the Narrow-Energy-Gap Organic Dye and Cosensitizer

A summary of the synthetic routes to organic dyes **H4** and **H15** is provided in Scheme S1. To avoid the use of toxic stannanes, 4-(2-ethylhexyl)-4*H*-dithieno­[3,2-*b*:2′,3′-*d*]­pyrrole (1) already reported in the literature^51^ was converted to 2-(7-bromobenzo­[*c*]­[1,2,5]­thiadiazol-4-yl)-4-(2-ethylhexyl)-4*H*-dithieno­[3,2-*b*:2′,3′-*d*]­pyrrole (**2**) at a yield of 51%, via a mild palladium-catalyzed direct arylation reaction. Compound **2** was then subjected to Suzuki–Miyaura cross-coupling with trianiline boronic acid pinacol ester **3**, furnishing the electron-rich compound **4** in a good yield of 73%. Next, **4** was treated with N-Bromosuccinimide to generate the monobrominated **5**. Immediately, the monobrominated **5** and compound **6**, via Suzuki–Miyaura cross-coupling reaction, afforded the aldehyde **7** with an excellent yield of 87%. In the last, in the presence of a weak base ammonium acetate, Knoevenagel condensation of aldehyde **7** and cyanoacetic acid gave the desired dye **H4**. Meanwhile, **H15** was obtained by a cross-coupling reaction and a hydrolysis reaction, at a total yield of 72%. Further information regarding the synthesis and structural characterization can be found in the Supporting Information.

### 2.2 Optical Properties, Energy Levels, and Optoelectronic Properties of Photosensitizers

Images of 0.1 mM dyes in dichloromethane are shown in Figure S4. The colors of the dye solution are dark blue for **H4**, and yellow for **H15**. As shown in Figure 1b, **H4** has a broad spectral response across the entire visible light region with a maximum absorption peak at 578 nm and a high molar extinction coefficient (*ε*) of 6.1 × 10^4^ M^–1^ cm^–1^ and a weak absorption around the blue light domain. To compensate for the light harvesting of **H4**, the cosensitizer **H15** has a strong absorption at 410 nm, with a good *ε* of 4.3 × 10^4^ M^–1^ cm^–1^. The UV–visible absorption spectra of both dyes and cosensitized **H4**/**H15** grafted-titania (TiO_2_) films are presented in Figure 1c. Compared with the **H4**-sensitized TiO_2_ film, the cosensitized film increased the absorption around 400 nm owing to the contribution of **H15**.

To elucidate the energy level alignments, cyclic voltammetry measurements were conducted on the two dyes grafted onto titania films within a three-electrode system, as shown in Figure 1d. The resulting first oxidation potentials (*E*_ox_) for **H4** and **H15** were determined to be 1.094 and 1.106 V vs SHE, respectively, which are more positive than the redox potential of [Co­(II)­(bpy)_3_]­[TFSI]_2_ (0.56 V vs SHE), ensuring an adequate driving force for hole injection. The zero–zero exciton energies (*E*_0–0_) determined from the intersection of the tangent absorption wavelength dyes on titania films (Figure 1b) are 1.70 eV for **H4**, and 2.38 eV for **H15**. The reduction potentials (*E*_red_) of the two dyes are more negative than the conduction band edge of TiO_2_ (−0.5 V vs SHE), being −0.61 V and −1.27 V vs SHE for **H4** and **H15** (Figure 1d), respectively. The corresponding energy level data can be found in Table S1.

### 2.3 Optoelectronic Properties of Photosensitizers and Device Performance

In order to gain information about the injection process, the time constants (τ) of time-resolved photoluminescence (PL) decays of **H4** and **H15** grafted onto semiconductor oxide films were determined by the femtosecond fluorescence up-conversion technique.^52–54^ Processed data are provided in Table S2, and for analytical details, see our previous work.^16^ As Figure 2a and b shows, the fluorescence kinetic traces of dye-grafted titania or alumina films differ significantly. Specifically, it is found that the excited-state lifetime of **H4** on alumina films is 97 ps, which is considerably shorter than that of 784 ps for **H15** alumina films. However, when grafted onto titania, much faster decay kinetics were observed for both dyes, with excited-state lifetimes reduced to 14.8 ps for **H4** on titania and 50.4 ps for **H15** (Figure 2c). This trend aligns with the energy gap law, which predicts that **H4** with its narrower energy gap is expected to exhibit faster radiative decay and shorter excited-state lifetimes due to thermally activated decay pathways.^55^

**Figure 2:**
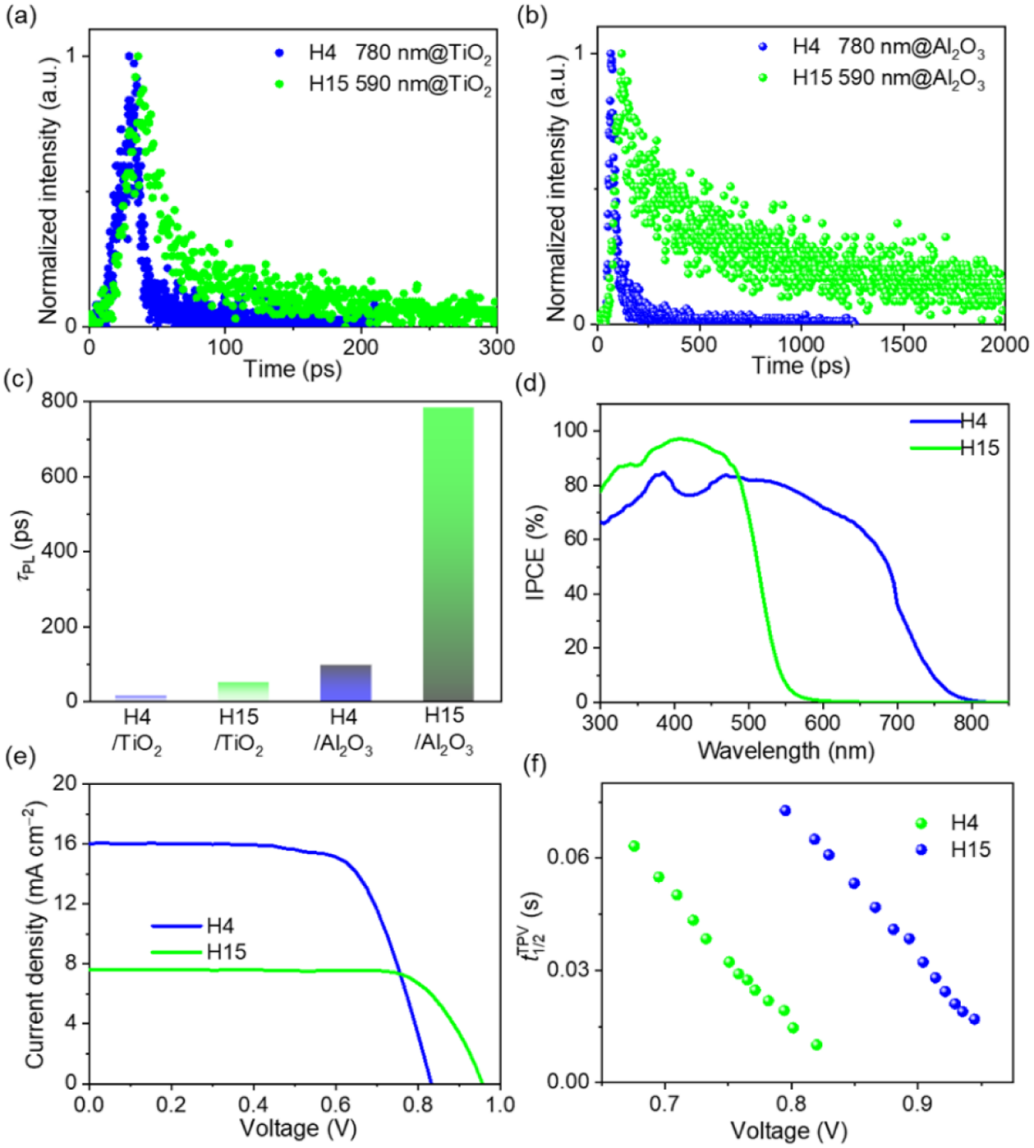
(a, b) Normalized ultrafast kinetic traces at selected photoluminescence (PL) wavelengths of **H4** and **H15** grafted on semiconductor oxide films: (a) TiO_2_, and (b) Al_2_O_3_. Excitation wavelength: 550 nm for **H4** films, and 410 nm for **H15** films. (c) Photoluminescence lifetime (τ_PL_) of **H4** and **H15** adsorbed on oxide films. (d) Plots of incident photon-to-electron conversion efficiency (IPCE) as a function of wavelength. (e) Current density–voltage (*J*–*V*) curves at an irradiance of the 100 mW cm^–2^, AM1.5G conditions. (f) Plots of half-lifetimes of electrons stored in titania as a function of voltage. The electron half lifetime $\left(t_{1/2}^{TPV}\right)$ is defined as the time required for the small-pulse transient photovoltage to decay to half of its initial value.

For solar cell fabrication, 4-μm-thick mesoporous transparent titania layers and 4-μm-thick mesoporous light-scattering titania layers were subjected to dyeing. A primrose yellow tris­(2,2′-bipyridine) cobalt (Co-bpy)-based redox electrolyte served as the electrolyte. The electrolyte recipes and cell fabrication are presented in the Experimental Section. Short-circuit incident photon-to-electron conversion efficiency (IPCE) measurements were performed by using monochromatic light in 5 nm wavelength increments (Figure 2d). The DSC based on **H4** attained a peak IPCE of 81%, which is substantially lower than the 95% value recorded for the **H15**-based device. This disparity in IPCE maxima is inferred to result from differences in the excited-state lifetimes of the respective dye-grafted titania films.

The photovoltaic performance of the DSCs sensitized with **H4** and **H15** was characterized by measuring the density–voltage (*J*–*V*) curves under standard AM1.5 illumination (100 mW cm^–2^), as depicted in Figure 2e. Table S3 provides a detailed summary of the photovoltaic parameters. Owing to the wide light-harvesting of AM1.5G photons, the **H4** cell exhibits a short-circuit photocurrent density (*J*_sc_) of 16.06 mA cm^–2^, an open-circuit photovoltage (*V*_oc_) of 833 mV, and a fill factor (FF) of 69.2%, yielding a good PCE of 9.25%. In sharp contrast, the **H15** cell owns a lower *J*_sc_ of 7.63 mA cm^–2^, but it possesses a very enhanced *V*_oc_ of 957 mV, affording a PCE of 5.56%. It is well known that the devices with copper electrolytes usually can achieve high *V*_oc_. Therefore, we fabricated devices with **H4** and **H15**. Interestingly, **H15** devices prepared with the copper electrolyte achieve a high *V*_oc_ value of 1.22 V (Figure S5, Table S4). Further, we performed transient photovoltage decay (TPV)^56,57^ measurements to reveal the interfacial energetics and dynamic properties. The electron half-lifetime $\left(t_{1/2}^{TPV}\right)$ , defined as the time required for the photovoltage to decrease to half of its initial value, is presented in Figure 2f. The half-lifetimes measured for **H15** are more than twice those observed for **H4**, a difference that arises in part from the superior interfacial charge recombination suppression capability of **H15**.

### 2.4 Efficient Charge Transfer and Interfacial Passivation Based on IBA

The incorporation of IBA into the cobalt electrolyte induces multiple changes from the cascade reaction. We have simply introduced these multifunctions in the above discussion. Now, we are going to systematically conduct research and discuss efficient oxidation and interaction. Figure 3a illustrates the multi-interaction from the cascade reaction of IBA in DSC. The molecule **H4** exhibits a typical donor–acceptor (D–A) structure, featuring strong π-conjugation through units such as triphenylamine (TPA), dithienopyrrole (DTP), thiophene, and benzothiadiazole. As shown in Figure 3b, **H4** shows an original absorption maximum at 578 nm with an absorption edge around 720 nm, indicating efficient light harvesting in the visible range. However, upon oxidation, a significant red shift is observed with the maximum absorption peak moving to 600 nm and the absorption tail extending beyond 1000 nm into the near-infrared region. This spectral evolution can be attributed to the oxidation-induced formation of radical cations, partially TPA•^+^ species, likely developing a new low-energy path transitions from the singly occupied molecular orbital (SOMO) to the lowest unoccupied molecular orbital (LUMO) (Figure S6). At the initial process, the TPA•^+^ facilitates intramolecular charge transfer and hole extraction (Figure S7). The oxidation process exceeds intramolecular charge transfer (ICT), resulting in a reduced energy gap with the value of 1.57 eV estimated from the UV–vis absorption (Figure 3b). Moreover, we recorded the time evolution of the in situ UV–vis measurements of the **H4**-sensitized TiO_2_ film exposed to the IBA-containing electrolyte. The characteristic absorption band of **H4** gradually shifts with the exposure time until stabilization, indicating that the dye is partly oxidized by IBA (Figure S8).

**Figure 3:**
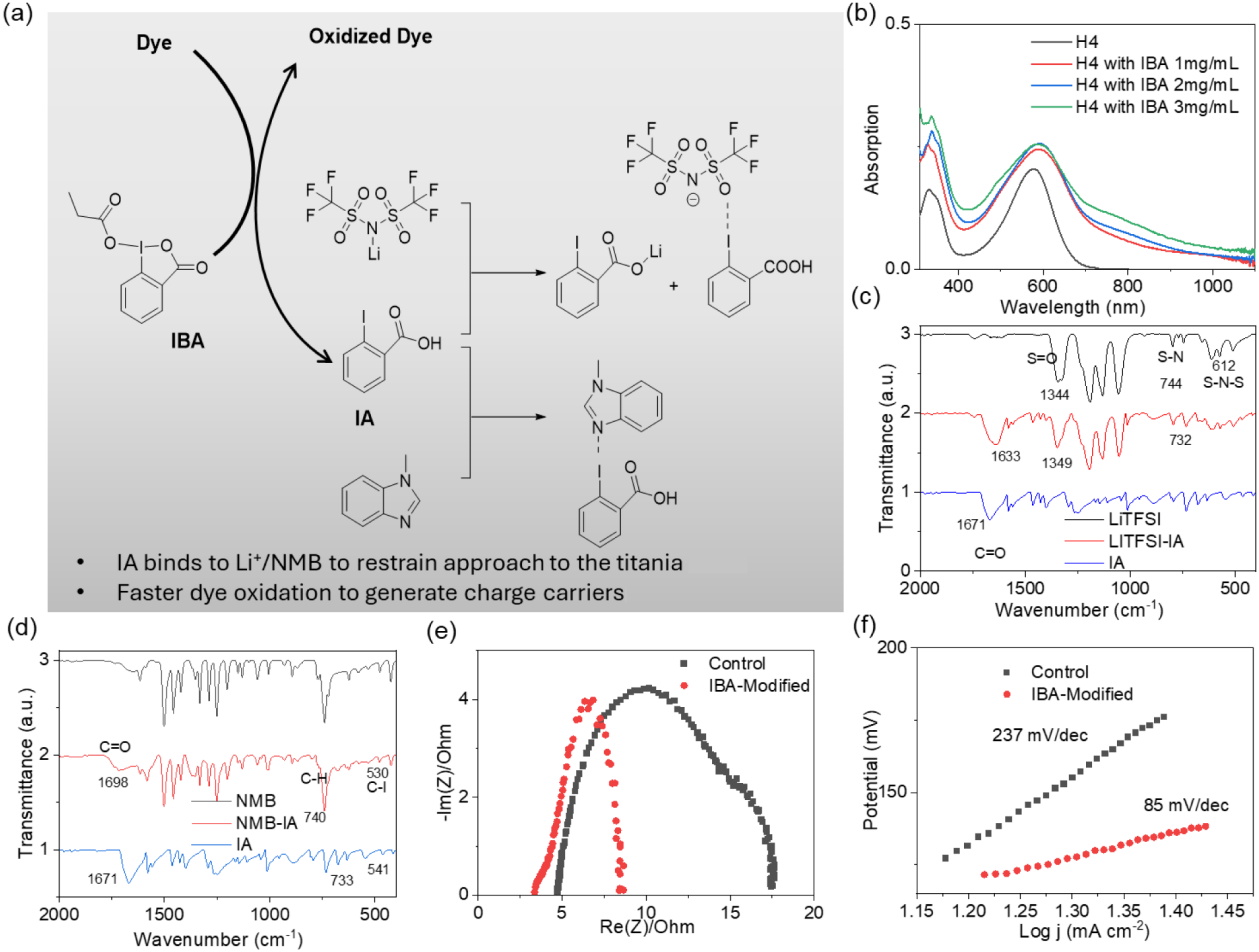
(a) The schematic diagram of multifunctions from cascade reaction of IBA in DSC. (b) UV–visible absorption spectra of **H4** solutions doped with different amounts of IBA. (c, d) Fourier transform infrared spectroscopy (FTIR) spectra of LiTFSI, NMB, IA, and the complex. The complex, LiTFSI, and NMB are measured in the solid state. The preparation of the complex involves dissolving the substance in acetonitrile and then evaporating it to dryness. (e) Impedance analysis: Nyquist plots of symmetrical cells with a cobalt electrolyte and iodine-doped cobalt electrolyte. (f) Linear sweep voltammetry (LSV) curves of symmetrical cells at a scan rate of 5 mV s^–1^. According to the Butler–Volmer formula, the Tafel equation is η = *a* + *b* log­(*i*).

Fourier transform infrared spectroscopy (FTIR), shown in Figure 3c, reveals that upon complexation with LiTFSI, the C=O stretching vibration of the carboxyl group in 2-iodobenzoic acid shifts significantly from 1671 cm^–1^ to 1633 cm^–1^, indicating coordination between the carbonyl oxygen and Li^+^ ions.^50^ Meanwhile, characteristic peaks of the TFSI^–^ anion also exhibit notable changes: the S–N stretching peak shifts from 744 cm^–1^ to 732 cm^–1^, and the S–N–S bending peak broadens from 612 cm^–1^, suggesting a decrease in anion symmetry and the formation of complex structures. Additionally, the S=O stretching vibration exhibits a slight blue shift from 1344 cm^–1^ to 1349 cm^–1^, implying perturbation of the electronic environment of TFSI^–^. These spectral changes collectively demonstrate the presence of multiple intermolecular interactions, including Li^+^ coordination, hydrogen bonding, and anion complexation between 2-iodobenzoic acid and LiTFSI.

In contrast, after interaction with NMB, presented in Figure 3d, the C=O stretching vibration peak of IA exhibits a blue shift from 1671 cm^–1^ to 1698 cm^–1^, indicating disruption of the carboxylic acid hydrogen-bonding network and strengthening of the C=O bond. The out-of-plane bending vibration of the aromatic C–H shifts slightly from 733 cm^–1^ to 740 cm^–1^, which may be attributed to π–π stacking interactions between aromatic rings. Furthermore, the C–I stretching vibration peak shifts from 541 cm^–1^ to 530 cm^–1^, suggesting weakening of the C–I bond likely due to halogen bonding (I···N) formed between the iodine atom and the nitrogen atom of the imidazole ring. These spectral features confirm the formation of stable noncovalent interactions between IA and NMB.

To further investigate the influence of the electrolyte additives on the interfacial processes at the counter electrode, we have performed electrochemical impedance spectroscopy (EIS)^58,59^ measurements on symmetrical device (CE//CE) with cobalt electrolyte and IBA additive electrolyte (Figure 3e). When comparing the Nyquist plots, we observed that the IBA-based DSC shows a notably smaller low-frequency semicircle than that of the cobalt electrolyte DSC, which we assign to Nernst diffusion in the electrolyte. The results demonstrate that the IBA additive significantly affects the charge transfer resistance at the counter electrode interface. Further, we perform the linear sweep voltammetry (LSV) curves of symmetrical cells (Figure 3f). According to the Tafel equation: η = *a* + *b* log­(*i*), the small slope of the IBA-modified counterpart is associated with a fast charge transfer dynamics.

Overall, the multifunctional design and IA-mediated interfacial passivation suppress interfacial charge recombination through effective chemical bonds: halogen bonding (I···N) with NMB, Li^+^ coordination via the carboxyl C=O group, and anion complexation with TFSI^–^. These bonding interactions collectively suppress ion mobility, enhance charge transfer kinetics, prevent ion aggregation toward the titania surface, and improve the photovoltaic performance of the DSC.

### 2.5 Photovoltaic Performance of Cosensitized Solar Cells Based on IBA

By cografting the narrow-energy-gap dye **H4** with the powerful suppressing-interface-recombination cosensitizer **H15**, we construct a compact and robust mixed self-assembled molecular layer on TiO_2_. As shown in Figure 4a, we found that **H4** has a substantially higher dye loading (*c*_m_) of 2.9× 10^–8^ mol cm^–2^ μm^–1^ on the surface of the mesoscopic TiO_2_ film compared with 2.1 × 10^–8^ mol cm^–2^ μm^–1^ for **H15**, while the dye load of the cosensitized film is 4.5 × 10^–8^ mol cm^–2^ μm^–1^. We determined the dye loading amounts by following the procedure described in the literature.^17^ The compact and ordered mixed self-assembled **H4**/**H15** layer significantly retards the recapture of TiO_2_ conduction-band electrons by a redox electrolyte, as shown below. To address both the issue of the required fast oxidation and the unfavorable migration of species from the electrolyte, we rationally incorporate IBA into the cobalt electrolyte (name it IBA-Modified) to produce multifunctions through the cascade reaction. Subsequently, we used compact cosensitized films to make solar cells with cobalt electrolyte (name it Control) or IBA-Modified. Figure 4b shows the *J–V* curve of the typical **H4**/**H15** cosensitized cell under standard AM1.5G, 100 mW cm^–2^ conditions. The parameters are listed in Table S3. In comparison to the **H4**-alone-based device, the PCE of the control cell is greatly improved to 11.76% (*V*_oc_ = 860 mV, *J*_sc_ = 18.13 mA cm^–2^, and FF= 0.755). The IBA-Modified device shows a higher PCE of 12.84%, corresponding to an enhanced *V*_oc_ of 885 mV, a slightly elevated *J*_sc_ of 18.32 mA cm^–2^, and an enlarged FF of 0.792. The incident photon-to-current efficiency (IPCE) spectra of the devices are presented in Figure 4d. The IBA-modified device exhibits consistently higher IPCE values than the control device over the wavelength range of 430–670 nm. Specifically, the peak IPCE value of the IBA-modified device reaches approximately 88% at around 500 nm, which is slightly higher than that of the control device. The integrated current density is consistent with the value from the *J–V* curves. Figure S9 shows a set of statistical results obtained from 20 individual solar cells of the Control devices and IBA-Modified devices, demonstrating good performance reproducibility. Moreover, the comparison of the present system with reported highly efficient DSCs using other dyes is detailed in Table S5.

**Figure 4:**
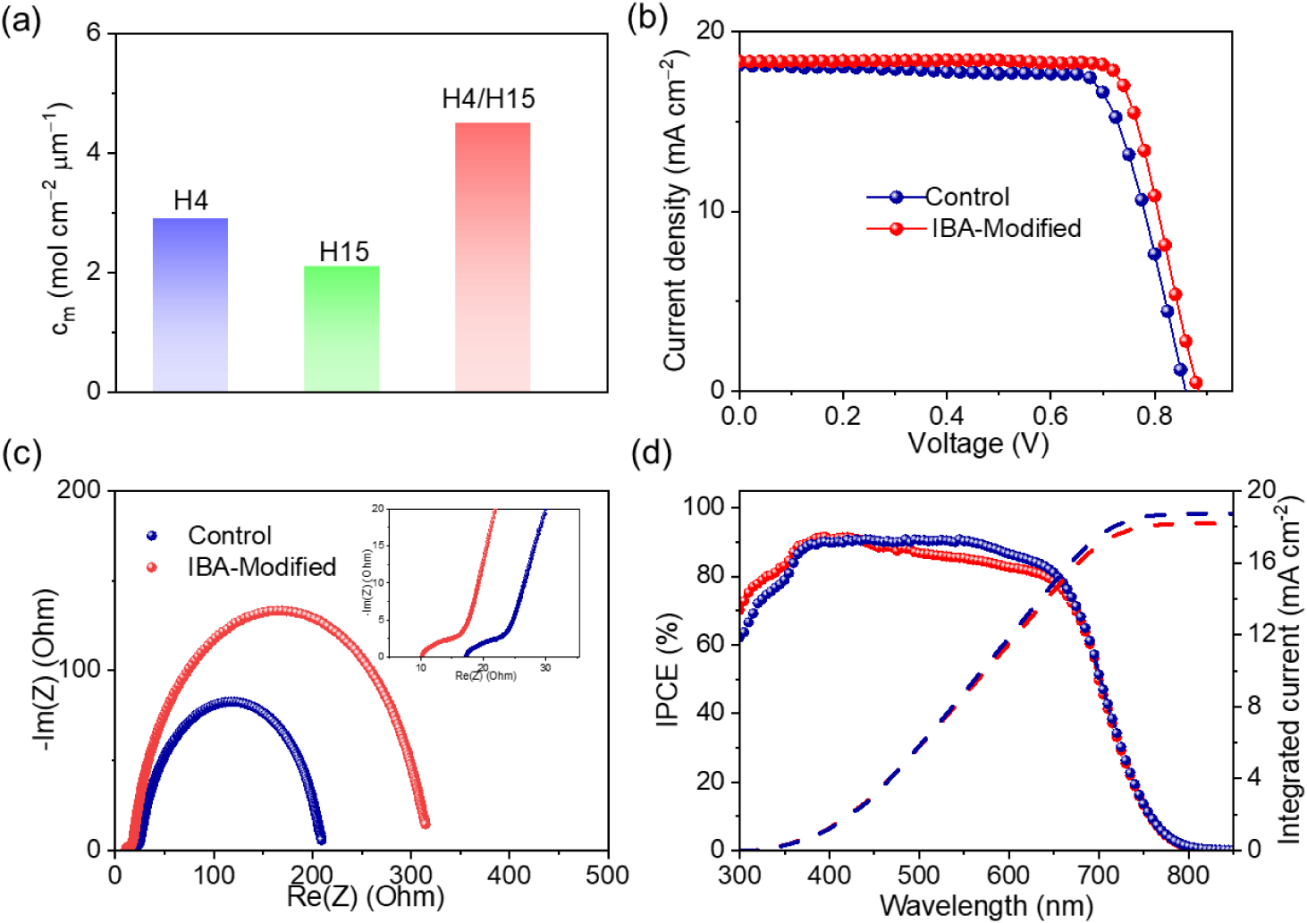
(a) Dye-loading amount (*c*_m_) on the surface of mesoporous TiO_2_ films. (b) Current density–voltage (*J–V*) curves of **H4**/**H15** cosensitized solar cells under the standard AM1.5G, 100 mW cm^–2^ conditions. (c) Impedance analysis: Nyquist plots under a forward bias of 0.86 V. The inset picture is the values at high frequencies. (d) Plots of incident photon-to-electron conversion efficiency (IPCE) and integrated current density as a function of wavelength of **H4**/**H15** cosensitized cells.

To move beyond simple chemical identification and understand the specific functional effects of these species, we have fabricated a new control device where the pure identified reduction byproduct IA was deliberately added to the electrolyte. As shown in Figure S10, the new control device exhibited a lower *V*_oc_ and a lower FF compared to the pristine IBA device (IBA-Modified). This suggests that free IA, when not part of a dynamic redox cycle, may adsorb onto the TiO_2_ surface and exacerbate recombination, unlike the IBA species, which is involved in a cascade reaction. The effectiveness of the hypercovalent iodide IBA in cobalt electrolyte has been proved improving the photovoltaic performance and reproducibility of DSCs. This modification approach is expected to be functional on copper electrolytes that are most widely used recently. For this, we incorporated IBA into the copper electrolyte in a DSC based on the XY1b dye (Figure S11). As for the DSC with XY1b-IBA (incorporated with IBA, Modified) achieved a high PCE of 11.31%, with a *J*_sc_ of 15.17 mA cm^–2^, an FF of 75.0%, and a *V*_oc_ of 0.994 V. As a comparison, the DSC with XY1b only achieved a PCE of 10.0%.

Electrochemical impedance spectroscopy was used to investigate the effect of introducing multifunctional IBA in the electrolyte on the charge transfer and transport processes of both DSC devices. Nyquist plots of the devices are presented in Figure 4c, whereas the schematic circuit is shown in Figure S12. The fitting parameters, including charge transfer resistance (*R*_ct_), chemical capacitance (*C*_μ_), charge recombination resistance (*R*_rec_), electron lifetime (τ_*n*_), and charge collection yields (η_cc_) can be found in Table S6. In Figure 4c, the first semicircle illustrates the transport resistance (*R*_ct_) that occurs at the PEDOT electrode–electrolyte interface in the high-frequency range. The second semicircle depicts charge transfer at the TiO_2_/dyes/electrolyte interface in the middle-frequency range. The corresponding charge recombination resistance (*R*_rec_) is in the order IBA-Modified > Control. A larger *R*_rec_ has lower recombination between the Co­(III) and electron in TiO_2_, leading to a higher *V*_oc_. A longer electron lifetime (τ_*n*_) of the IBA-Modified device indicates less electron recombination, which increases *V*_oc_. Overall, the IBA modification reduces interfacial charge recombination and enhances charge transfer at the cathode. These improvements contribute to the superior photocurrent generation efficiency of the IBA-modified device.

### 2.6 Long-Term Stability of Solar Cells

Indoor photovoltaics are promising alternatives to traditional energy storage devices to supply low-power Internet of Things (IoT) ecosystem-based devices.^19^ In recent years, cobalt-based electrolytes have been widely investigated for indoor DSSCs owing to their high open-circuit voltage and low charge recombination. Lee et al. reported cobalt-based indoor DSCs with PCE surpassing 26% by using polymeric counter electrodes.^23^ Kim et al. employed polymer gel electrolytes with cobalt complex, achieving remarkable PCEs of 27.5% under 1000 lx CFL light.^24^ Herein, we measured the photovoltaic performance of a modified device with a hypervalent additive under light-emitting diode (LED) irradiance, simulating indoor light. We show the typical *J–V* curves in Figure S13 and summarize the photovoltaic performance parameters for the modified **H4/H15** device in Table S7, measured under different LED light intensities. At a light intensity of 4500 lx (1.33 mW cm^–2^) from the LED lamp, the modified **H4/H15** device displays a *V*_oc_ of 791 mV, a *J*_sc_ of 0.556 mA cm^–2^ and an FF of 78.1%, yielding a power density of 0.343 mW cm^–2^ and the corresponding good PCE of 25.81%, which is significantly superior to many state-of-the-art cobalt-mediated indoor DSSCs, highlighting the advantages of the optimized electrolyte and device structure.

Long-term stability under indoor light soaking is a critical requirement for the practical deployment of DSCs. Figure 5a–d represents the evolution of photovoltaic parameters of the cobalt electrolyte-based DSCs aged in indoor light soaking for 1000 h. It is noteworthy that the IBA-Modified cell owes excellent stability with a mere 5% drop in its initial PCE, which is much better than that of 22% for the control device (Figure 5d). As is commonly reported, a decline in *V*_oc_ is evident across all aged devices, posing a substantial limitation to the overall PCE. The IBA-modified cell exhibits a *V*_oc_ loss of only ∼51 mV, while the control device suffers a larger drop of ∼91 mV after 1000 h (Figure 5a). Notably, the short-circuit current density (*J*_sc_) of the IBA-modified cell remains effectively constant, whereas the control cell displays a reduction of ∼0.64 mA cm^–2^ (Figure 5b). The fill factor (FF) remains stable in the IBA-modified device but shows a modest decline in the control device (Figure 5c). Electron half-lifetime $\left(t_{1/2}^{TPV}\right)$ , derived from the transient photovoltage decay as depicted in Figure 5e, decreases after 1000 h of light soaking, potentially leading to a reduced quasi-Fermi level in TiO_2_ and consequently a lower *V*_oc_. Based on these observations, we propose a degradation mechanism illustrated in Figure 5f. The presence of IBA in the electrolyte enables coordination with Li^+^ ions, effectively mitigating their accumulation at the TiO_2_ surface. This suggests that long-term stability in DSCs can be enhanced by minimizing the direct interaction between the TiO_2_ surface and the electrolyte—achievable through the design of supramolecular assemblies and interfacial passivation, which inhibits Li^+^ interfacial access.

**Figure 5:**
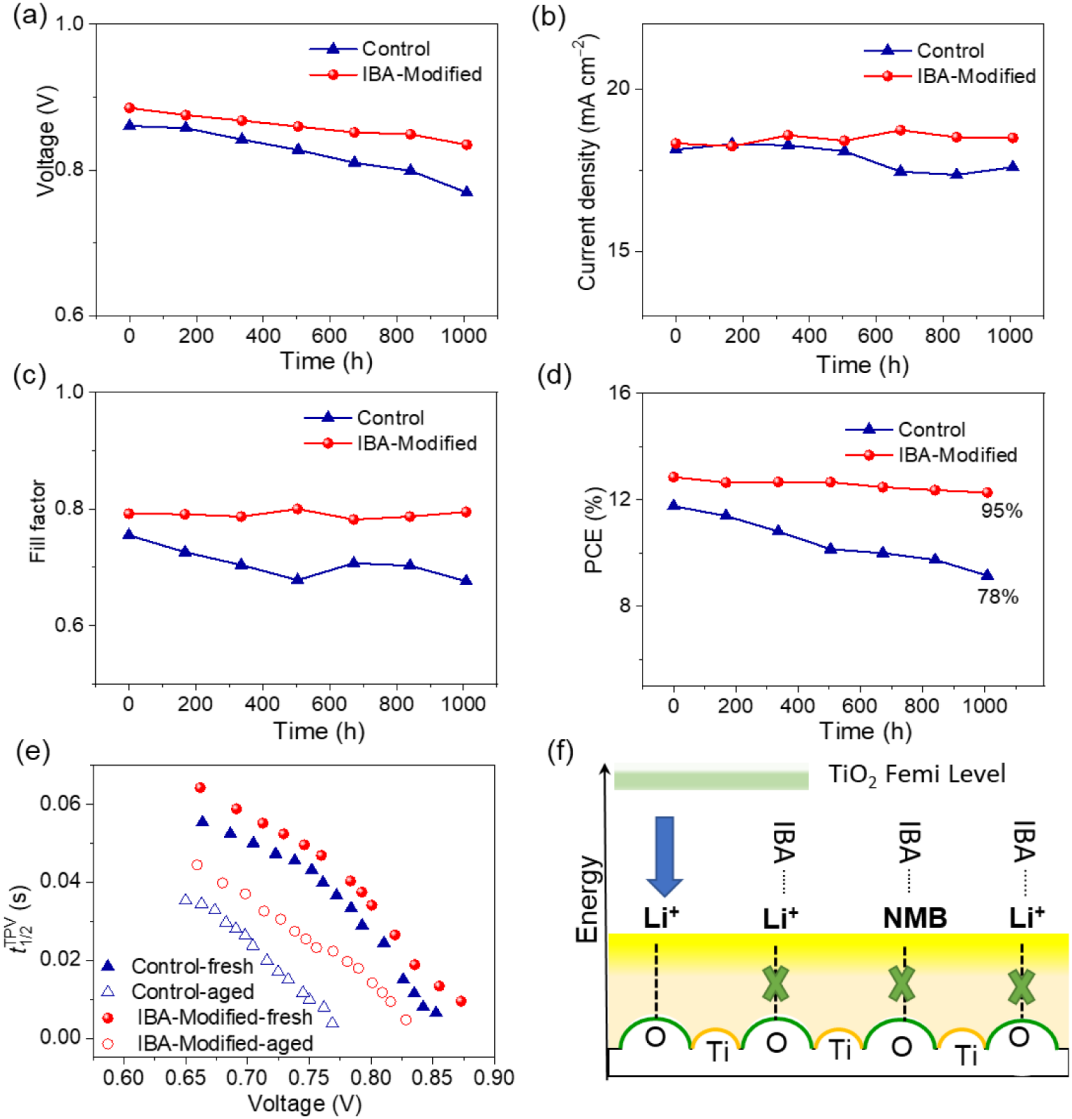
Evolution of photovoltaic parameters of the control device and the IBA-Modified device aged in the indoor light and electrical analyses. (a–d) Open-circuit Photovoltage (a), Current density (b), Fill factor (c), and power conversion efficiency (PCE) (d). (e) Comparison of electron lifetimes of fresh and aged devices measured with the small-pulse transient photovoltage decay method against voltage. (f) Proposed mechanism for the degradation of thermally aged cells.

## 3 Conclusions

In summary, we have demonstrated a synergistic strategy to enhance both the power conversion efficiency and long-term operational stability of cobalt-based dye-sensitized solar cells (DSCs) through the dual implementation of cosensitization and electrolyte engineering. The newly synthesized cosensitizer **H15**, characterized by a prolonged excited-state lifetime, complements the spectral response of the narrow-energy-gap dye **H4**, thereby broadening the light absorption range and suppressing interfacial recombination in cosensitized titania film. Concurrently, the incorporation of 1-acetoxy-1,2-benziodoxol-3­(1*H*)-one (IBA), a hypervalent iodine­(III) compound, into the cobalt electrolyte generates free radicals and accelerates charge transfer. The redox byproduct 2-iodobenzoic acid (IA) not only restricts Li^+^ migration through carbonyl coordination but also forms halogen bonds with electrolyte additives, collectively mitigating charge recombination. This multifunctional design and IA-mediated interfacial passivation enable a good power conversion efficiency of 12.84%, with only minor degradation observed after 1000 h under indoor light soaking. Our results highlight the potential of combining molecular engineering and electrolyte innovation to overcome key bottlenecks in cobalt-based DSC technology, paving the way for the future development of efficient and durable solar energy devices.

## 4 Experimental Section

### 4.1 Materials

Acetonitrile, ethanol, and chloroform were distilled and then used. LiTFSI, NMB, and FC were purchased from Sigma-Aldrich and used without further purification. The synthetic details of **H4** and **H15** are provided in the Electronic Supporting Information.

### 4.2 Fabrication of Solar Cells

The detailed mesoporous TiO_2_ and PEDOT counter electrode fabrication procedure can be found in the literature.^21^ After sintering at 500 °C in air for 30 min and cooling down to 90 °C, the mesoporous TiO_2_ was immersed into a 0.1 mM corresponding dye solution for 14 h at 25 °C for dye uptake. The dye solution of **H15** was prepared by dissolving 0.1 mM **H15** in chloroform/ethanol (*v*/*v*, 1/19). The dye solution of **H4** was made by dissolving 0.1 mM **H4** and 0.5 mM chenodeoxycholic acid in CF/EtOH (*v*/*v*, 2/8). The solution for cosensitization of **H4**/**H15** was prepared by dissolving 0.1 mM **H4**, 0.1 mM **H15**, and 0.5 mM chenodeoxycholic acid in CF/EtOH (*v*/*v*, 2/8). The dye-grafted titania electrode and PEDOT electrode were pressed together mechanically without a spacer and further sealed with UV light-curing glue (UV RESIN), which is quickly solidified by UV light (Alonefire SV41 UV Flashlight). The cobalt electrolyte was injected into the sealed electrodes through a predrilled hole on the counter electrode, completing the fabrication of the sandwich-type solar cell. The control device is in combination with tris­(2,2′-bipyridine)­cobalt (Co-bpy)-based redox electrolyte, and the recipe is 0.2 M Co­(II)­(bpy)_3_(TFSI)_2_, 0.1 M Co­(III)­(bpy)_3_(TFSI)_3_, 0.1 M lithium bis­(trifluoromethanesulfonyl)­imide (LiTFSI), and 0.6 M *N*-methylbenzimidazole (NMB) in acetonitrile. The IBA-modified device incorporates 2 mg mL^–1^ of IBA into the Co-bpy electrolyte.

### 4.3 Cyclic Voltammetry and Electrochemical Impedance Measurements

The electrochemical characterization of titania oxide surface-grafted dyes was conducted by using a BioLogic SP150e potentiostat in a classical three-electrode configuration with electrochemical cyclic voltammetry (CV) procedures. A dye adsorbed on a 2-μm-thick transparent mesoscopic TiO_2_ film served as the working electrode, while Ag/AgCl and platinum wire acted as the reference and counter electrodes, respectively. A 0.1 M Tetrabutylammonium hexafluorophosphate (TBAPF_6_) solution was utilized as the supporting electrolyte, and the scan rate was set to 50 mV/s. The ferrocene/ferrocenium redox system functioned as an internal standard to calibrate the quasi-reference electrode before and after the measurements. Impedance measurements were performed using a BioLogic SP150e potentiostat, over a frequency range from 1 MHz down to 0.1 Hz at a bias potential between 0 and 0.86 V (with a 40 mV sinusoidal AC perturbation).^58,59^ All of the EIS measurements were carried out in the dark (room temperature). The results of the impedance spectra were analyzed with the EC-Lab V11.52 Program. According to the Nyquist plots, we can obtain the key physical parameters: charge transfer resistance (*R*_ct_), chemical capacitance (*C*_μ_), charge recombination resistance (*R*_rec_), electron lifetime (τ_*n*_), and charge collection yields (η_cc_), whereas the electron lifetime (τ) can be imparted by eq 1

1 $$\tau_{n}=R_{rec}\times C_{\mu }$$

The charge collection efficiencies (η_cc_) are given by eq 2

2 $$\eta_{\mathrm{cc}}=1-\frac{R_{tr}}{R_{tr}+R_{rec}}$$

### 4.4 Photophysical Characterizations and Data Analysis

The static UV–vis spectra were analyzed using a UV-2401PC spectrometer (Agilent). Time-resolved photoluminescence (PL) measurements were performed using a femtosecond fluorescence up-conversion setup integrated with a streak camera system (Hamamatsu, C10910-05), enabling subpicosecond temporal resolution. The system captures ultrafast emission dynamics from dye-sensitized samples with high temporal and spectral accuracy. The fundamental femtosecond laser pulses (920 nm, 6160 mW) were generated by a Ti:sapphire regenerative amplifier (Astrella, Coherent Inc.). The output was split into two beams: one central portion was directed into an optical parametric amplifier (OPA) to produce the excitation (pump) pulse at 530 nm. The detailed operation process and data fitting can be found in our previous work.^16^

### 4.5 Device Characterization

Current density–voltage (*J–V*) characteristics were measured using an ABET 11000 solar simulator and a Keithley 2400 source meter under standard 1 Sun illumination (100 mW cm^–2^, AM 1.5G), which was calibrated with a Si reference cell. The scan rate was 0.1 V/s, and the active device area was 0.08 cm^2^. Incident photon-to-current efficiency (IPCE) was measured using BENTHAM’s BenWin^+^ Spectral Acquisition Software. Transient photovoltage (TPV) measurements were conducted using a white LED driven by a programmable power supply and a control box for switching between open- and short-circuit states. Voltage signals were recorded with a Yokogawa DLM2052 oscilloscope, and light perturbation pulses (520 nm) were provided by an Oxxius laser.

## Supplementary Material



## Notes

Details molecular synthesis procedure and additional figures and tables, NMR spectra, ATR-FTIR spectra, and mass spectra (PDF)
