# Supplementary material for: Synergistic Cosensitization and Redox-Triggered Interfacial Engineering for Efficient and Durable Solar Cells
Source: ACS Appl Mater Interfaces. 2026 Apr 28;18(18):26252–62. doi: 10.1021/acsami.6c01394 (PMC13181725; doi:10.1021/acsami.6c01394)
Supplement: Supplementary file 1 [file am6c01394_si_001.pdf]

Supporting Information

Synergistic Co-Sensitization and Redox-Triggered Interfacial Engineering for Efficient and Durable Solar Cells

Heng Wu,*[ad] Laia Marín Moncusí,[ab] Javier Perez Hernandez,[a] Eugenia Martinez-Ferrero[a] and Emilio Palomares*[ac]

[a]*Institute of Chemical Research of Catalonia (ICIQ)-CERCA, Avinguda Països Catalans, 16, Tarragona, 43007, Spain*

[b]*Universitat Rovira i Virgili (URV), Departament D'enginyeria electrònica Elèctrica i Automàtica, Avinguda Països Catalans, 26, Tarragona, Spain*

[c]*Catalan Institution for Research and Advanced Studies (ICREA), Passeig Lluïs Companys, 23, Barcelona, Spain*

[d]*School of Chemistry and Chemical Engineering, Key Laboratory of Electrochemical Energy Storage and Energy Conversion of Hainan Province, Key Laboratory of Electrochemical Energy Storage and Light Energy Conversion Materials of Haikou City, Hainan Normal University, Haikou 571158, China*

**E-mail: hwu@iqic.es; epalomares@iciq.es*

EXPERIMENTAL SECTION

Materials. Lithium bis(trifluoromethanesulfonyl)imide (LiTFSI), *N*-methylbenzimidazole (NMB), 1-ethyl-3-methylimidazolium bis(trifluoromethanesulfonyl)imide (EMITFSI), ferrocene (Fc), bis(pinacolato)diboron, tetrabutylammonium hexafluorophosphate ($TBAPF_6$), *N*-Bromosuccinimide (NBS), [1,1'-bis(diphenylphosphino)ferrocene]dichloropalladium(II) ($Pd(dppf)Cl_2$), tetrakis(triphenylphosphine)palladium ($Pd(PPh_3)_4$), palladium(II) acetate ($Pd(OAc)_2$), 2-(2,6-dimethoxybiphenyl)-dicyclohexylphosphine (Sphos), tricyclohexylphosphine tetrafluoroborate ($PCy_3{\cdot}HBF_4$), pivalic acid (PivOH), potassium carbonate (K_2CO_3), potassium acetate, (KOAc), potassium hydroxide (KOH), potassium phosphate (K_3PO_4), 4,7-dibromobenzo[c][1,2,5]thiadiazole, (5-formylthiophen-2-yl)boronic acid, ammonium acetate, 2-iodobenzoic acid (IA), and hydrochloric acid were purchased from Sigma-Aldrich. Toluene, chloroform, dioxane, and tetrahydrofuran (THF), 3-methoxypropionitrile were dried and distilled before use. 4-(2-ethylhexyl)-4H-dithieno[3,2-b:2',3'-d]pyrrole[S1] (1), *N*-(2',4'-bis(hexyloxy)-[1,1'-biphenyl]-4-yl)-2',4'-bis(hexyloxy)-*N*-(4-(4,4,5,5-tetramethyl-1,3,2-dioxaborolan-2-yl)phenyl)-[1,1'-biphenyl]-4-amine[S2] (3), ethyl 4-(7-bromo-2-(2-ethylhexyl)-2H-benzo[d][1,2,3]triazol-4-yl)benzoate[S3] (8), and 1-Acetoxy-1,2-benziodoxol-3(1H)-one (IBA)[S4] were synthesized according to the respective literature procedures. Other chemical agents were purchased and used without further purification. The photosensitizing dyes **H4** and **H15** were prepared according to Scheme S1.

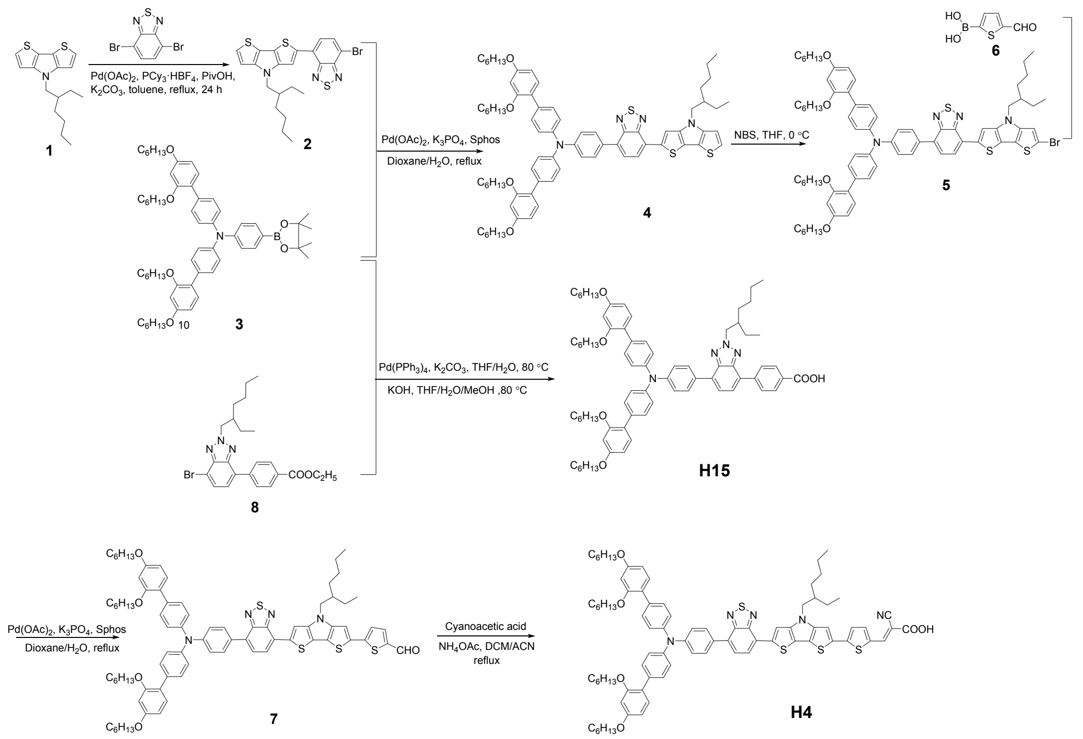

Scheme S1. Synthetic procedures of dyes **H4** and **H15**.

Synthesis of 2-(7-bromobenzo[c][1,2,5]thiadiazol-4-yl)-4-(2-ethylhexyl)-4H-dithieno[3,2-b:2',3'-d]pyrrole (**2**): In a dried Schlenk tube were dissolved 4-(2-ethylhexyl)-4H-dithieno[3,2-b:2',3'-d]pyrrole (1) (730 mg, 2.5 mmol), ethyl 4-(7-bromobenzo[c][1,2,5]thiadiazol-4-yl)benzoate (736 mg, 3.0 mmol), K_2CO_3 (518 mg,

3.7 mmol) in toluene (10 mL). Then $Pd(OAc)_2$ (28 mg, 0.125 mol), $PCy_3{\cdot}HBF_4$ (92 mg, 0.25 μmol), and PivOH (77 mg, 0.75 mol) were added to the reaction mixture in a nitrogen-filled atmosphere, which was refluxed for 24 h. The mixture was extracted three times with chloroform before the organic phase was washed with water and dried over anhydrous sodium sulfate. After solvent removal under reduced pressure, the crude product was purified by column chromatography (dichloromethane/hexane, 1/2, *v*/*v*) on silica gel to yield a red solid as the desired product **2** (620 mg, 51% yield). ^1H NMR (400 MHz, $CDCl_3$) δ 8.30 (d, *J* = 1.1 Hz, 1H), 7.82 (dd, *J* = 7.7, 1.3 Hz, 1H), 7.68 (dd, *J* = 7.8, 1.3 Hz, 1H), 7.23 (d, *J* = 5.3 Hz, 1H), 7.01 (d, *J* = 5.3 Hz, 1H), 4.15 (qd, *J* = 14.4, 7.3 Hz, 2H), 2.03 (p, *J* = 6.4 Hz, 1H), 1.49 – 1.26 (m, 9H), 0.94 (dt, *J* = 22.6, 7.4 Hz, 6H). ^{13}C NMR (101 MHz, $CDCl_3$) δ 153.97, 151.70, 146.34, 146.05, 135.57, 132.38, 128.41, 124.59, 124.33, 115.72, 114.87, 113.27, 111.09, 111.03, 51.29, 40.51, 30.66, 28.67, 24.14, 22.99, 14.10, 10.75, 1.03.

Synthesis of N-(2',4'-bis(hexyloxy)-[1,1'-biphenyl]-4-yl)-N-(4-(7-(4-(2-ethylhexyl)-4H-dithieno[3,2-b:2',3'-d]pyrrol-2-yl)benzo[c][1,2,5]thiadiazol-4-yl)phenyl)-2',4'-bis(hexyloxy)-[1,1'-biphenyl]-4-amine (**4**): 2-(7-bromobenzo[c][1,2,5]thiadiazol-4-yl)-4-(2-ethylhexyl)-4H-dithieno[3,2-b:2',3'-d]pyrrole (200 mg, 0.4 mmol), N-(2',4'-bis(hexyloxy)-[1,1'-biphenyl]-4-yl)-2',4'-bis(hexyloxy)-N-(4-(4,4,5,5-tetramethyl-1,3,2-dioxaborolan-2-yl)phenyl)-[1,1'-biphenyl]-4-amine (440 mg, 0.48 mmol), $Pd(OAc)_2$ (4.5 mg,0.02 mmol), Sphos (8.1 mg, 0.02 mmol), K_3PO_4 (126 mg, 0.6 mmol) and dioxane/H_2O (10 mL, *v*/*v*, 5/1) were added to a three-neck round-bottom flask under argon. After the reaction mixture was refluxed for 5 h, the solution was cooled to room temperature, brine (10 mL) was added into the solution, and the mixture was extracted by CH_2Cl_2 (20 mL × 3). After the combined organic phase was dried over Na_2SO_4, the solvent was removed under reduced pressure and the residue was purified by silica gel column chromatography (dichloromethane/hexane, *v*/*v*, 1/2) to give the desired product (352 mg, 73%). ^1H NMR (400 MHz, $CDCl_3$) δ 8.36 (s, 1H), 7.98 – 7.90 (m, 3H), 7.73 (d, *J* = 7.6 Hz, 1H), 7.55 – 7.47 (m, 4H), 7.38 – 7.19 (m, 9H), 7.03 (d, *J* = 5.3 Hz, 1H), 6.62 – 6.55 (m, 4H), 4.26 – 4.11 (m, 2H), 4.01 (dt, *J* = 7.8, 6.5 Hz, 8H), 2.07 (q, *J* = 6.5 Hz, 1H), 1.82 (ddd, *J* = 15.2, 8.7, 6.5 Hz, 8H), 1.58 – 1.27 (m, 33H), 0.94 (ddt, *J* = 18.6, 9.0, 7.3 Hz, 18H). ^{13}C NMR (101 MHz, $CDCl_3$) δ 159.59, 157.01, 148.10, 146.02, 145.58, 136.86, 133.44, 130.89, 130.61, 130.29, 129.77, 127.30, 126.94, 124.76, 124.23, 124.02, 123.03, 122.91, 115.22, 112.53, 111.09, 105.36, 100.47, 68.45, 68.14, 51.31, 40.53, 31.64, 31.48, 30.69, 29.34, 29.11, 28.70, 25.79, 24.16, 23.02, 22.64, 22.60, 14.12, 14.07, 10.77.

Synthesis of N-(2',4'-bis(hexyloxy)-[1,1'-biphenyl]-4-yl)-N-(4-(7-(6-bromo-4-(2-ethylhexyl)-4H-dithieno[3,2-b:2',3'-d]pyrrol-2-yl)benzo[c][1,2,5]thiadiazol-4-yl)phenyl)-2',4'-bis(hexyloxy)-[1,1'-biphenyl]-4-amine (**5**): Compound **4** (316 mg, 0.26 mmol) was dissolved in THF (20 mL), and the solution was cooled to 0 °C using an ice salt bath. NBS (46 mg, 0.26 mmol) in THF (5 mL) was dropwised to the reaction mixture. Then the resulting solution was stirred at 0 °C for 0.5 h. Water was added to terminate the reaction and the mixture was extracted by CH_2Cl_2 (20 mL × 3). After the combined organic phase was dried over Na_2SO_4, the solvent was removed under reduced pressure and the residue was purified by silica gel column chromatography (toluene/hexane, *v*/*v*, 1/2) to give the product **5**. The crude product **5** was used to synthesized compound **7** without further purification.

Synthesis of 5-(6-(7-(4-(bis(2',4'-bis(hexyloxy)-[1,1'-biphenyl]-4-yl)amino)phenyl)benzo[c][1,2,5]thiadiazol-4-yl)-4-(2-ethylhexyl)-4H-dithieno[3,2-b:2',3'-d]pyrrol-2-

yl)thiophene-2-carbaldehyde (**7**): Compound **5** (580 mg, 0.45 mmol), compound **6** (139 mg,0.9 mmol), $Pd(OAc)_2$ (5 mg, 0.023 mmol), Sphos (9.2 mg, 0.023 mmol), K_3PO_4 (142 mg, 0.67 mmol) and dioxane/H_2O (10 mL, *v*/*v*, 5/1) were added to a three-neck round-bottom flask under argon. After the reaction mixture was refluxed overnight, the solution was cooled to room temperature, brine (10 mL) was added into the solution and the mixture was extracted by CH_2Cl_2 (20 mL × 3). After the combined organic phase was dried over Na_2SO_4, the solvent was removed under reduced pressure and the residue was purified by silica gel column chromatography (DCM/hexane, *v*/*v*, 1/2) to give product **7** (510 mg, 86%). ^1H NMR (500 MHz, $CDCl_3$) δ 9.85 (s, 1H), 8.29 (s, 1H), 7.96 – 7.88 (m, 3H), 7.71 (d, *J* = 7.6 Hz, 1H), 7.65 (d, *J* = 4.0 Hz, 1H), 7.55 – 7.49 (m, 4H), 7.35 – 7.29 (m, 4H), 7.28 – 7.23 (m, 6H), 6.60 – 6.56 (m, 4H), 4.14 (qd, *J* = 14.5, 7.3 Hz, 2H), 4.01 (dt, *J* = 9.0, 6.5 Hz, 8H), 2.11 – 1.94 (m, 1H), 1.86 – 1.71 (m, 8H), 1.52 – 1.32 (m, 32H), 1.00 – 0.89 (m, 18H). ^{13}C NMR (101 MHz, $CDCl_3$) δ 182.18, 159.61, 157.01, 154.11, 152.68, 148.86, 148.23, 147.27, 145.68, 145.51, 140.72, 139.15, 137.56, 133.53, 132.12, 130.90, 130.31, 129.79, 127.09, 126.37, 125.09, 124.30, 123.12, 122.99, 122.75, 116.56, 115.17, 112.10, 109.58, 105.36, 100.47, 68.45, 68.14, 51.32, 40.50, 31.64, 31.49, 30.64, 29.35, 29.11, 28.64, 25.80, 25.78, 24.17, 23.02, 22.65, 22.60, 14.13, 14.07, 10.78.

Synthesis of (E)-3-(5-(6-(7-(4-(bis(2',4'-bis(hexyloxy)-[1,1'-biphenyl]-4-yl)amino)phenyl)benzo[*c*][1,2,5]thiadiazol-4-yl)-4-(2-ethylhexyl)-4H-dithieno[3,2-b:2',3'-d]pyrrol-2-yl)thiophen-2-yl)-2-cyanoacrylic acid (**H4**) :Compound 8 (110 mg, 0.083 mmol) and cyanoacetic acid (281 mg, 3.3 mmol) dissoved in acetonitrile/ chloroform (18 mL, *v*/*v*, 1/2) were added to a three-neck round-bottom flask. Then ammonium acetate (5.7 mg, 0.074 mmol) was added to the reaction mixture, which was stirred at reflux for 48 h. After the solution was cooled to room temperature, the mixture was extracted by CH_2Cl_2 (20 mL × 3). The organic phase was washed with 0.2 M hydrochloric acid solution and water in turn. Then the organic phase was dried over Na_2SO_4, the solvent was removed under reduced pressure and the residue was purified by silica gel column chromatography (dichloromethane/methanol, *v*/*v*, 20/1) to give product 105 mg (91%). ^1H NMR (400 MHz, $CDCl_3$) δ 8.03 (s, 1H), 7.73 (s, 1H), 7.54 (s, 3H), 7.35 (d, *J* = 7.4 Hz, 6H), 7.20 – 7.15 (m, 2H), 7.02 (d, *J* = 8.4 Hz, 6H), 6.85 (d, *J* = 16.3 Hz, 2H), 6.57 – 6.45 (m, 4H), 3.94 (dt, *J* = 20.7, 6.7 Hz, 10H), 1.98 (s, 1H), 1.85 – 1.68 (m, 8H), 1.48 – 1.26 (m, 32H), 1.01 – 0.84 (m, 18H). ^{13}C NMR (101 MHz, $CDCl_3$) δ 159.47, 156.92, 145.23, 133.28, 130.90, 130.13, 124.24, 122.95, 105.23, 100.40, 77.22, 68.36, 68.07, 31.67, 31.46, 29.38, 29.05, 25.81, 25.71, 23.15, 22.65, 22.57, 14.29, 14.07, 10.81. HR-MS m/z calcd. for ($C_{84}H_{95}N_5O_6S_4$): 1397.6165. Found: 1397.6159. IR (ν_{max}, cm^{-1}) 2923, 2853, 1673, 1598, 1569, 1516, 1490, 1467, 1409, 1382, 1321, 1260, 1223, 1180, 1053, 818. Melting point: 128.3 – 129.2°C.

Synthesis 4-(7-(4-(bis(2',4'-bis(hexyloxy)-[1,1'-biphenyl]-4-yl)amino)phenyl)-2-(2-ethylhexyl)-2H-benzo[d][1,2,3]triazol-4-yl)benzoic acid (**H15**):

Compound **3** (91 mg, 0.2 mmol), compound **8** (0.22 g, 0.24 mmol) and $Pd(PPh_3)_4$ (148.0 mg, 0.13 mmol) were dissolved in the mixture of THF (10 mL) and 2M K_2CO_3 solution (2.5 mL). After the reaction mixture was stirred at 80 °C for 48 h, the solution was cooled to room temperature, brine (50 mL) was added into the solution, and the mixture was extracted by CH_2Cl_2 (50 mL × 3). After the combined organic phase was dried over Na_2SO_4, the solvent was removed under reduced pressure and the residue was purified by silica gel column chromatography (hexane/dichloromethane, *v*/*v*, 1/1) to give yellow oil product (77%). In a

round-bottom flask were dissolved the desired ethyl ester (230 mg, 0.15 mmol) and KOH (171 mg, 3.0 mmol) in a solvent mixture of THF/H_2O (20 mL, *v*/*v*, 3/1). The reaction mixture was refluxed for 5 h and then cooled to room temperature. Chloroform was added before the organic phase was washed with 0.1 M hydrochloric acid and water in turn and then dried over anhydrous sodium sulfate. After solvent removal under reduced pressure, the crude product was purified by column chromatography (dichloromethane/methanol, 10/1, *v*/*v*) on silica gel to yield a yellow solid as the desired dye.(93%) ^{1}H NMR (400 MHz, $CDCl_3$) δ 8.29 (q, *J* = 8.6 Hz, 4H), 8.08 (d, *J* = 11.4 Hz, 2H), 7.82 – 7.65 (m, 2H), 7.60 – 7.46 (m, 4H), 7.41 – 7.29 (m, 4H), 6.59 (d, *J* = 7.0 Hz, 4H), 4.76 (d, *J* = 6.9 Hz, 2H), 4.02 (q, *J* = 6.8 Hz, 8H), 2.34 (p, *J* = 6.2 Hz, 1H), 1.82 (ddd, *J* = 15.0, 8.6, 6.4 Hz, 8H), 1.53 – 1.30 (m, 32H), 1.04 – 0.87 (m, 18H). ^{13}C NMR (101 MHz, $CDCl_3$) δ 171.57, 159.66, 157.04, 142.86, 130.57, 128.45, 128.11, 121.82, 105.35, 100.45, 77.24, 68.44, 68.15, 59.94, 40.42, 31.64, 31.48, 30.57, 29.72, 29.34, 29.10, 28.44, 25.80, 25.78, 23.99, 22.99, 22.65, 22.60, 14.07, 14.06, 10.55. HR-MS m/z calcd. for ($C_{75}H_{94}N_4O_6$): 1146.7173. Found: 1146.7164. IR (ν_{max}, cm^{-1}) 3055, 2954, 2931, 2661, 1687, 1602, 1493, 1467, 1422, 1265, 1182, 896, 829, 731, 703. Melting point: 76.2 – 77.1 °C.

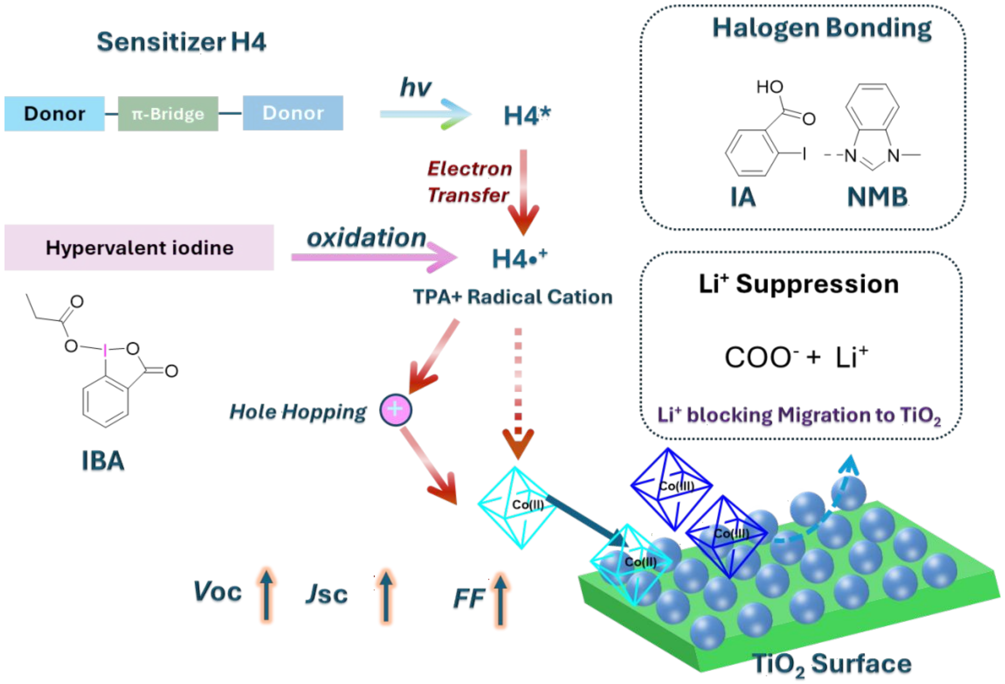

Figure S1. The molecular structures of 1-acetoxy-1,2-benziodoxol-3(1H)-one (IBA) and 2-iodobenzoic acid (IA).

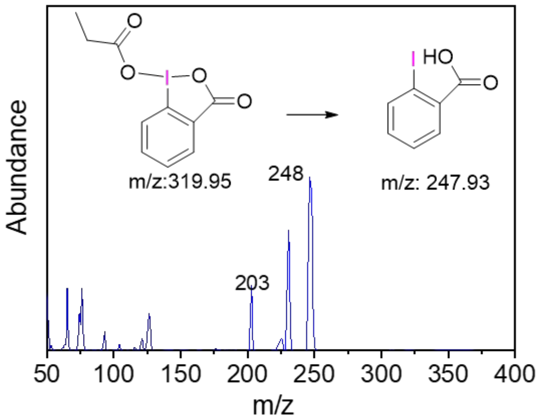

Figure S2. GC result of oxidation of H4 with 1-acetoxy-1,2-benziodoxol-3(1H)-one. The m/z peak value of 248 belongs to 2-iodobenzoic acid.

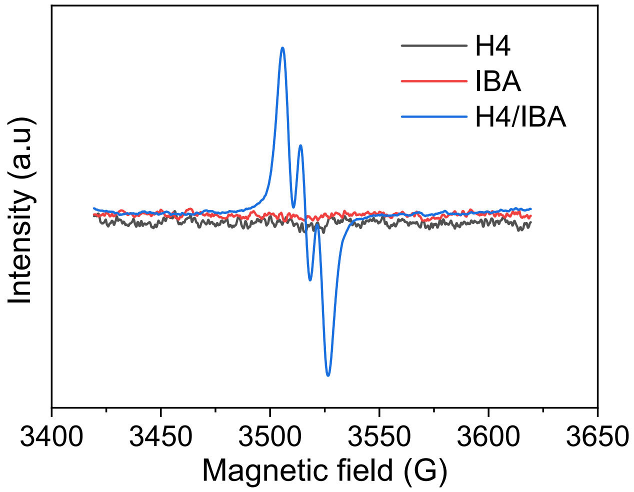

Figure S3. EPR data of dilute solution in chloroform of H4, IBA, and H4/IBA.

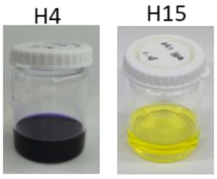

Figure S4. Pictures of dyes dissolved in dichloromethane (0.1 mM)

Table S1. Optical and electrochemical properties of dyes **H4** and **H15**.

Dyes	$\lambda_{abs,max}^{DCM}$ [a] (nm)	$\lambda_{abs,max}^{film}$ [b] (nm)	E_{0-0} [c] (eV)	E_{ox} [d] (V vs. SHE)	E_{red}^{*} [e] (V vs. SHE)
**H4**	578 (ε: 6.1 × 10^4 M^{-1} cm^{-1})	559	1.70	1.09	−0.61
**H15**	410 (ε: 4.3 × 10^4 M^{-1} cm^{-1})	415	2.38	1.10	−1.27

[a] Measured from 0.1 M dyes solution in dichloromethane (DCM), presented in Figure 1b. [b]Measured from dyes anchored on the 4.0 μm-thick transparent TiO_2 films, presented in Figure 1c. [c]Calculated from the absorption onset wavelength (λ_{onset}) of the corresponding dye using the formula: E_{0-0} = 1240 / λ_{onset}. The λ_{onset} determined from the intersection of the tangent absorption wavelength dyes on titania films. [d]Measured from the cyclic voltammogram of the corresponding dye adsorbed on a 4.0 μm-thick transparent TiO_2 film in a 0.1M tetrabutylammonium hexafluorophosphate (TBAPF6)/acetonitrile supporting electrolyte vs. ferrocene as internal standard, presented in Figure 1d. Then, the potential vs. ferrocene were converted to that vs. standard hydrogen electrode (SHE) by adding 0.624. [e]Calculated from formula: $E_{red} = E_{ox} - E_{0-0}$.

Table S2. Time constants and amplitudes employed to fit fluorescence decays of dye grafted alumina and titania films[a].

	**H4**@ Al_2O_3	**H4**@ TiO_2	**H15** @ Al_2O_3	**H15**@ TiO_2
τ_1 (ps)	15.7	6.2	64.6	12.4
τ_2 (ps)	122.5	22.1	868.7	75.3
A_1	0.71	0.75	0.61	0.80
A_2	0.29	0.25	0.39	0.20
$\bar{\tau}$ (ps)	97	14.8	784	50.4

[a] The amplitude-averaged lifetime of fluorescence at a certain wavelength was calculated with the equation $\bar{\tau} = \sum_{i=1}^{n} Ai\tau i / \sum_{i=1}^{n} Ai$ (A$i > 0$). . Excitation wavelength: 550 nm for **H4**, and 410 nm for **H15**.

Table S3. Photovoltaic parameters of solar cells with cobalt electrolyte measured under the standard AM1.5G sunlight.

Devices	$J_{sc}^{IPCE\ a}$ (mA cm^{-2})	J_{sc} (mA cm^{-2})	V_{oc} (mV)	FF (%)	PCE (%)
**H4**	15.97±0.13	16.06±0.15	833±4	69.2±0.2	9.25±0.2
**H15**	7.74±0.11	7.63±0.12	957±3	76.1±0.3	5.56±0.1
**H4/H15** control	18.18±0.14	18.13±0.16	860±2	75.5±0.2	11.76±0.2
**H4/H15** IBA-Modified	18.73±0.22	18.32±0.24	885±3	79.2±0.4	12.84±0.2

[a]was computed by wavelength integral of the samples of the IPCE measured at the short-circuit and the standard AM 1.5G emission spectrum. (BenWin+)

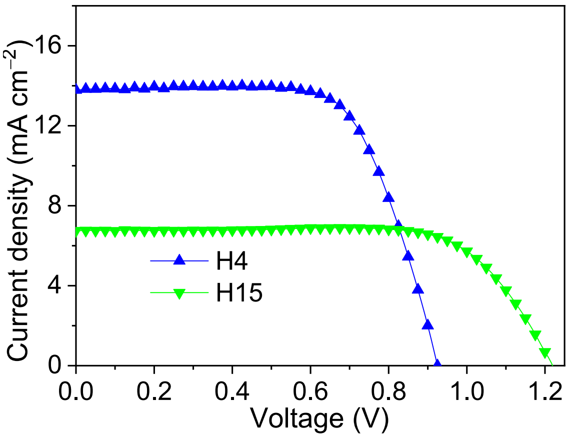

Figure S5. Current−voltage (*J*–*V*) curves of device with copper electrolyte at an irradiance of the 100 mW cm^{-2}, AM1.5G conditions. Electrolyte: 0.16 M [Cu(I)(tmby)$_2$]TFSI and 0.08 M [Cu(II)(tmby)$_2$](TFSI)$_2$ complexes with 0.1 M LiTFSI and 0.6 M NMB in acetonitrile.

Table S4. Photovoltaic parameters of solar cells with copper electrolyte measured under the standard AM1.5G sunlight.

Devices	J_{sc} (mA cm^{-2})	V_{oc} (mV)	FF (%)	PCE (%)
**H4**	13.82	924	68.8	8.79
**H15**	6.76	1217	72.5	5.97

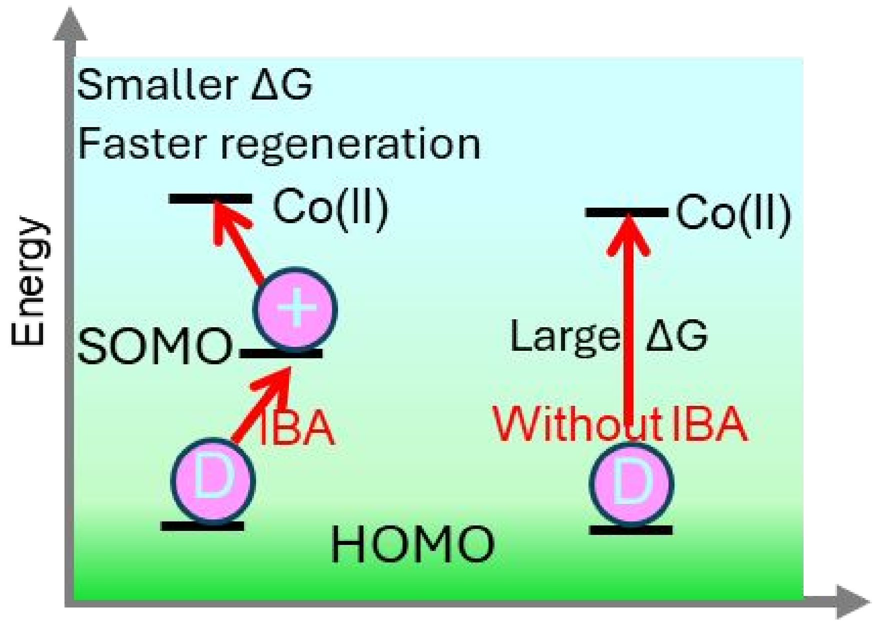

Figure S6. The schematic diagram of **H4** dye molecule oxidized by IBA and charge transfer process.

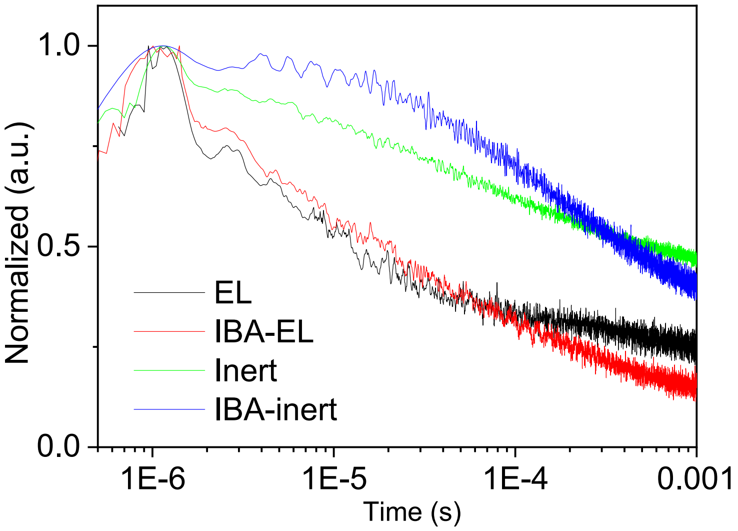

Figure S7. Transient absorption spectroscopy measurements of dyes-sensitized TiO_2 films immersed in a cobalt electrolyte (EL), a cobalt electrolyte with 2 mg mL^{-1} IBA (IBA-EL) or an inert electrolyte, an inert electrolyte with IBA (IBA-inert). Pulse fluence: 35 μJ cm^{-2}. Pump wavelength: 520 nm; probe wavelength: 800 nm. For the **H4/H15** co-sensitized transparent titania films in the presence of the inert electrolyte (0.6 M NMB and 0.1 M LiTFSI) or the IBA-inert electrolyte (2 mg mL^{-1} IBA added to the inert electrolyte), slow kinetic traces correlated with the interfacial charge recombination of photo-oxidized dye molecules with electrons in titania were observed in the millisecond time region, with half-reaction time constants of 439 μs for the inert electrolyte and 462 μs for the IBA-inert electrolyte. In contrast, due to the hole injection from the photo-oxidized dye molecules to cobalt (II) ions, fast kinetic decays of dyed titania films with cobalt electrolyte (EL) or IBA-modified cobalt electrolyte (IBA-EL, 2 mg mL^{-1} IBA added) occurred in the microsecond time domain. The half-reaction time constant was 20.6 μs for the EL sample, nearly double that of 11.7 μs for the IBA-EL counterpart, suggesting faster dye regeneration. Overall, the hole injection yield (ϕ_{hi}) for the IBA-modified sample was 97.5%, which is higher than the 95.3% estimated for the control device. These results demonstrate that the introduction of IBA facilitates rapid hole extraction through a faster oxidation process. We performed operation process and data fitting by following our previous work.[S20]

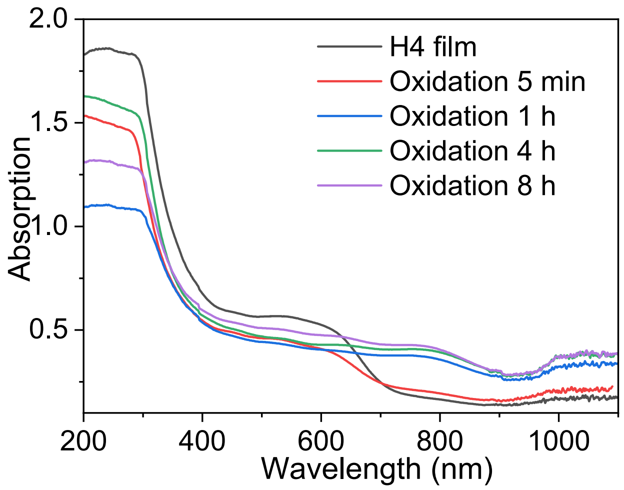

Figure S8. UV-visible absorption spectra of **H4**-grafted titania films immersed in a solution of 2 mg mL^{-1} IBA in acetonitrile with different immersing time.

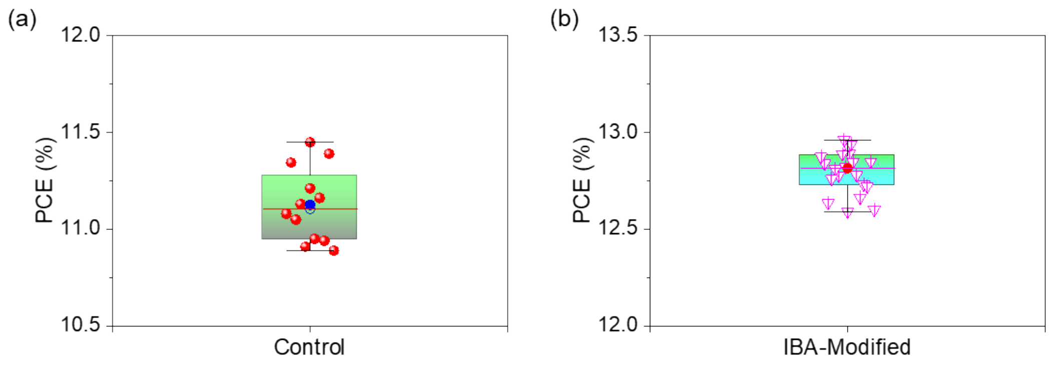

Figure S9. Statistics of PCE of the co-sensitized solar cells based on 20 samples of Control devices (a) and IBA-Modified devices with cobalt electrolyte (b).

Table S5. Photovoltaic performance of high-efficient DSSCs using various dyes under standard AM 1.5G conditions.

Dye	J_{sc} (mA cm^{-2})	V_{oc} (V)	FF (%)	PCE (%)	Redox shuttles	Refs
YD2-o-C8/Y123	17.66	935	74.0	12.3	$[Co(bpy)_3]^{2+/3+}$	S5
SM371	15.9	0.96	79.0	12.0	$[Co(bpy)_3]^{2+/3+}$	S6
SM315	18.1	0.91	78.0	13.0	$[Co(bpy)_3]^{2+/3+}$	S6
ADEKA-1	15.6	1.036	77.4	12.5	$[Co(phen)_3]^{2+/3+}$	S7
ADEKA-1/LEG4	18.27	1.014	77.1	14.3	$[Co(phen)_3]^{2+/3+}$	S8
ZL001	20.57	0.887	70.0	12.8	$[Co(bpy)_3]^{2+/3+}$	S9
ZL003	20.73	0.956	68.5	13.6	$[Co(bpy)_3]^{2+/3+}$	S9
SGT149/SGT021	20.86	0.912	73.2	13.9	$[Co(bpy)_3]^{2+/3+}$	S10
C293	17.28	0.974	74.7	12.6	$[Co(phen)_3]^{2+/3+}$	S11
C275	17.03	0.956	77.0	12.5	$[Co(phen)_3]^{2+/3+}$	S12
R6	19.69	0.850	75.4	12.6	$[Co(bpy)_3]^{2+/3+}$	S13
H4/H15	18.32	0.885	**79.2**	12.84	$[Co(bpy)_3]^{2+/3+}$	This work
C281	21.69	0.815	73.5	13.0	$[Co(bpy)_3]^{2+/3+}$	S14
R7/Y123	16.15	1.035	76.1	12.7	$[Cu(tmby)_2]^{1+/2+}$	S15
HY64	15.76	1.025	77.4	12.5	$[Cu(tmby)_2]^{1+/2+}$	S16
ZS4	16.3	1.05	77.1	13.2	$[Cu(tmby)_2]^{1+/2+}$	S17
XY1b/MS5	15.84	1.05	81.3	13.5	$[Cu(tmby)_2]^{1+/2+}$	S18
SL9/SL10	17.8	1.04	82.1	15.2	$[Cu(tmby)_2]^{1+/2+}$	S19
XY1b/H7	15.89	1.051	82.1	13.7	$[Cu(tmby)_2]^{1+/2+}$	S20

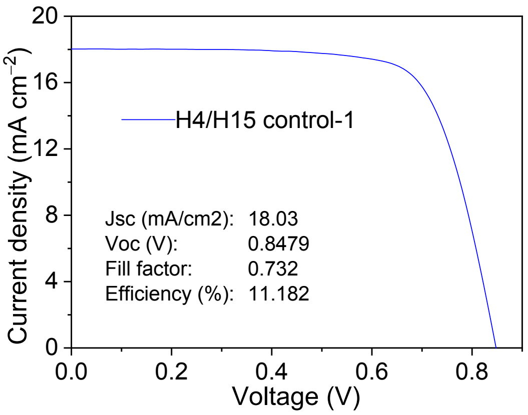

Figure S10. Current–voltage (*J*–*V*) curves of **H4/H15** cosensitized devices with a new control cobalt electrolyte, by directly adding 2-iodobenzoic acid (IA) (2 mg mL^{-1}) to the cobalt electrolyte, at the same molar concentration as the IBA used in the standard device.

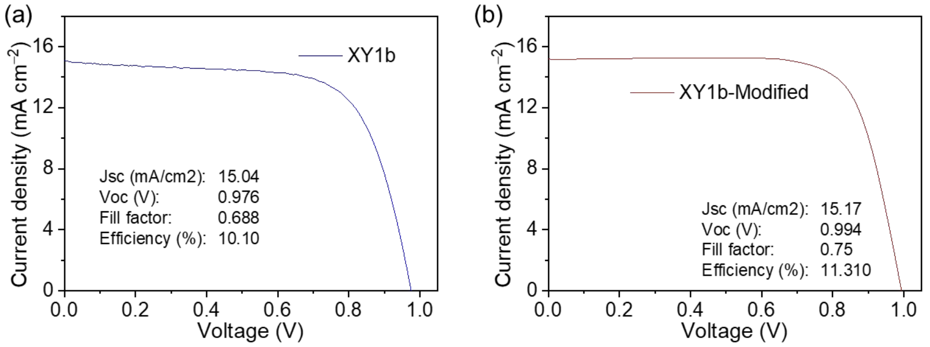

Figure S11. Current–voltage (*J–V*) curves of XY1b devices in combination with copper electrolyte (a) and hypercovalent iodine dopant-copper electrolyte (Modified) (b). Electrolyte recipe: 0.16 M $[Cu(I)(tmby)_2]TFSI$ and 0.08 M $[Cu(II)(tmby)_2](TFSI)_2$ complexes with 0.1 M LiTFSI and 0.6 M NMB in acetonitrile. The IBA-modified device was added 2 mg mL^{-1} hypercovalent iodine into the copper electrolyte.

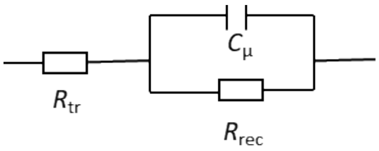

Figure S12. Equivalent circuit of electrochemical impedance spectroscopy.

Table S6. EIS parameters for **H4**/**H15** co-sensitized solar cells with/without IBA.

Devices	R_{ct} (Ω)	R_{rec} (Ω)	C_{μ} (mF)	τ (ms)	η_{cc}
Control	15.2	173	0.1217	21.1	91.9%
IBA-Modified	13.3	270	0.1049	28.4	95.4%

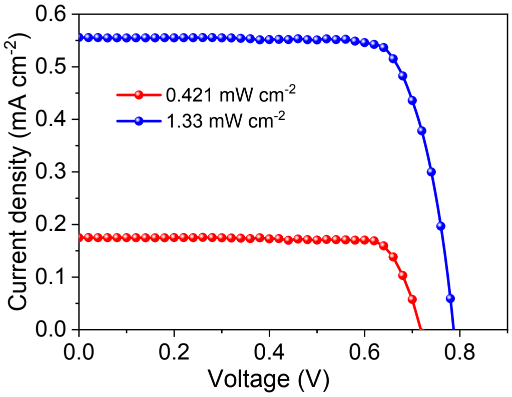

Figure S13. *J–V* curves for the modified-device **H4/H15** recorded at illuminated LED light.

Table S7. Photovoltaic parameters of the modified-device **H4/H15** measured at a LED light illumination.

Illuminance (lux)	Irradiance (mW cm^{-2})	P_{out} (mW cm^{-2})	J_{sc} (mA cm^{-2})	V_{oc} (mV)	FF (%)	PCE (%)
1500	0.421	0.1048	0.175	778	76.9	24.87
4500	1.33	0.343	0.556	791	78.1	25.81

REFERENCES

(S1) Förtsc, S.; Vogt, A.; Bäuerle, P. New Methods for the Synthesis of 4*H*-Dithieno[3,2-*b*:2′,3′-*d*]Pyrrole. *J. Phys. Org. Chem.* **2017**, *30*, e3743.

(S2) Gao, P.; Kim, Y. J.; Yum, J.-H.; Holcombe, T. W.; Nazeeruddin, M. K.; Grätzel, M. Facile Synthesis of a Bulky BPTPA Donor Group Suitable for Cobalt Electrolyte Based Dye Sensitized Solar Cells. *J. Mater. Chem. A* **2013**, *1*, 5535–5541.

(S3) Ren, Y.; Zhang, D.; Suo, J.; Cao, Y.; Eickemeyer, F. T.; Vlachopoulos, N.; Zakeeruddin, S. M.; Hagfeldt, A.; Grätzel, M. Hydroxamic Acid Pre-Adsorption Raises the Efficiency of Cosensitized Solar Cells. *Nature* **2023**, *613*, 60–65.

(S4) Declas, N.; Waser, J. Access to Vinyl Ethers and Ketones with Hypervalent Iodine Reagents as Oxy-Allyl Cation Synthetic Equivalents. *Angew. Chem. Int. Ed.*, 2020, **59**, 18256–18260.

(S5) Yella, A.; Lee, H.-W.; Tsao, H. N.; Yi, C.; Chandiran, A. K.; Nazeeruddin, M. K.; Diau, E. W.-G.; Yeh, C.-Y.; Zakeeruddin, S. M.; Grätzel, M. Porphyrin-Sensitized Solar Cells with Cobalt (II/III)–Based Redox Electrolyte Exceed 12 Percent Efficiency. *Science* **2011**, *334*, 629–634.

(S6) Mathew, S.; Yella, A.; Gao, P.; Humphry-Baker, R.; Curchod, B. F. E.; Ashari-Astani, N.; Tavernelli, I.; Rothlisberger, U.; Nazeeruddin, M. K.; Grätzel, M. Dye-Sensitized Solar Cells with 13% Efficiency Achieved through the Molecular Engineering of Porphyrin Sensitizers. *Nat. Chem.* **2014**, *6*, 242–247.

(S7) Kakiage, K.; Aoyama, Y.; Yano, T.; Otsuka, T.; Kyomen, T.; Unno, M.; Hanaya, M. *Chem. Commun.* **2014**, *50*, 6379–6381.

(S8) Kakiage, K.; Aoyama, Y.; Yano, T.; Oya, K.; Fujisawa, J.; Hanaya, M. Highly-Efficient Dye-Sensitized Solar Cells with Collaborative Sensitization by Sily-Anchor and Carboxy-Anchor Dyes. *Chem. Commun.* **2015**, *51*, 15894–15897.

(S9) Zhang, L.; Yang, X.; Wang, W.; Gurzadyan, G. G.; Li, J.; Li, X.; An, J.; Yu, Z.; Wang, H.; Cai, B.; Hagfeldt, A.; Sun, L. 13.6% Efficient Organic Dye-Sensitized Solar Cells by Minimizing Energy Losses of the Excited State. *ACS Energy Lett*. **2019**, *4*, 943−951.

(S10) Ji, J.; Zhou, H.; Eom, Y. K.; Kim, C. H.; Kim, H. K. 14.2% Efficiency Dye-Sensitized Solar Cells by Co-sensitizing Novel Thieno[3,2-b]indole-Based Organic Dyes with a Promising Porphyrin Sensitizer. *Adv. Energy Mater*. **2020**, *10*, 2000124.

(S11) Wang, J.; Wu, H.; Jin, L.; Zhang, J.; Yuan, Y.; Wang, P. *Chemsuschem*., **2017**, *10*, 2962−2967.

(S12) Yao, Z.; Zhang, M.; Wu, H.; Yang, L.; Li, R.; Wang, P. Donor/Acceptor Indenoperylene Dye for Highly Efficient Organic Dye-Sensitized Solar Cells. *J. Am. Chem. Soc*. **2015**, *137*, 3799–3802.

(S13) Ren, Y.; Sun, D.; Cao, Y.; Tsao, H. K.; Yuan, Y.; Zakeeruddin, S. M.; Wang, P.; Grätzel, M. A Stable Blue Photosensitizer for Color Palette of Dye-Sensitized Solar Cells Reaching 12.6% Efficiency. *J. Am. Chem. Soc.* **2018**, *140*, 2405–2413.

(S14) Yao, Z.; Wu, H.; Li, Y.; Wang, J.; Zhang, J.; Zhang, M.; Guo, Y.; Wang, P. Dithienopicenocarbazole as the Kernel Module of Low-Energy-Gap Organic Dyes for Efficient Conversion of Sunlight to Electricity. *Energy Environ. Sci*. **2015**, *8*, 3192–3199.

(S15) Ren, Y.; Flores-Díaz, N.; Zhang, D.; Cao, Y.; Decoppet, J.; Fish, G.C.; Moser, J.E.; Zakeeruddin, S. M.; Wang, P.; Hagfeld, A.; Grätzel, M. Blue Photosensitizer with Copper(II/I) Redox Mediator for Efficient and Stable Dye-Sensitized Solar Cells. *Adv. Funct. Mater*. **2020**, 2004804.

(S16) Jiang, H.; Ren, Y.; Zhang, W.; Wu, Yong.; Socie, E. C.; Carlsen, B. I.; Moser, J.; Tian, H.; Zakeeruddin, S. M.; Zhu, W-H.; Grätzel, M. Phenanthrene-Fused-Quinoxaline as a Key Building Block for Highly Efficient and Stable Sensitizers in Copper-Electrolyte-Based Dye-Sensitized Solar Cells. *Angew. Chem. Int. Ed*. **2020**, *59*, 9324–9329.

(S17) Grobelny, A.; Shen, Z.; Eickemeyer, F. T.; Antariksa, N. F.; Zapotoczny, S.; Zakeeruddin, S. M.; Grätzel, M. A Molecularly Tailored Photosensitizer with an Efficiency of 13.2% for Dye Sensitized Solar Cells. *Adv. Mater.* **2023**, *35*, 202207785.

(S18) Zhang, D.; Stojanovic, M.; Ren, Y.; Cao, Y.; Eickemeyer, F. T.; Socie, E.; Vlachopoulos, N.; Moser, J.-E.; Zakeeruddin, S. M.; Hagfeldt, A.; Grätzel, M. A Molecular Photosensitizer Achieves a Voc of 1.24 V Enabling Highly Efficient and Stable Dye-Sensitized Solar Cells with Copper(II/I)-Based Electrolyte. *Nat. Commun*. **2021**, *12*, 1777.

(S19) Ren, Y.; Zhang, D.; Suo, J.; Cao, Y.; Eickemeyer, F. T.; Vlachopoulos, N.; Zakeeruddin, S. M.; Hagfeldt, A.; Grätzel, M. Hydroxamic Acid Pre-Adsorption Raises the Efficiency of Cosensitized Solar Cells. *Nature* **2023**, *613*, 60–65.

(S20) Wu, H.; Moncusí, L. M.; Li, J.; Martinez-Ferrero, E.; Wang, P.; Palomares. E.; *Adv. Sci.* **2025**, *12*, e09116.

APPENDIX: ^{1}H NMR, ^{13}C NMR, mass Spectra, and ATR-FTIR spectra of new compounds

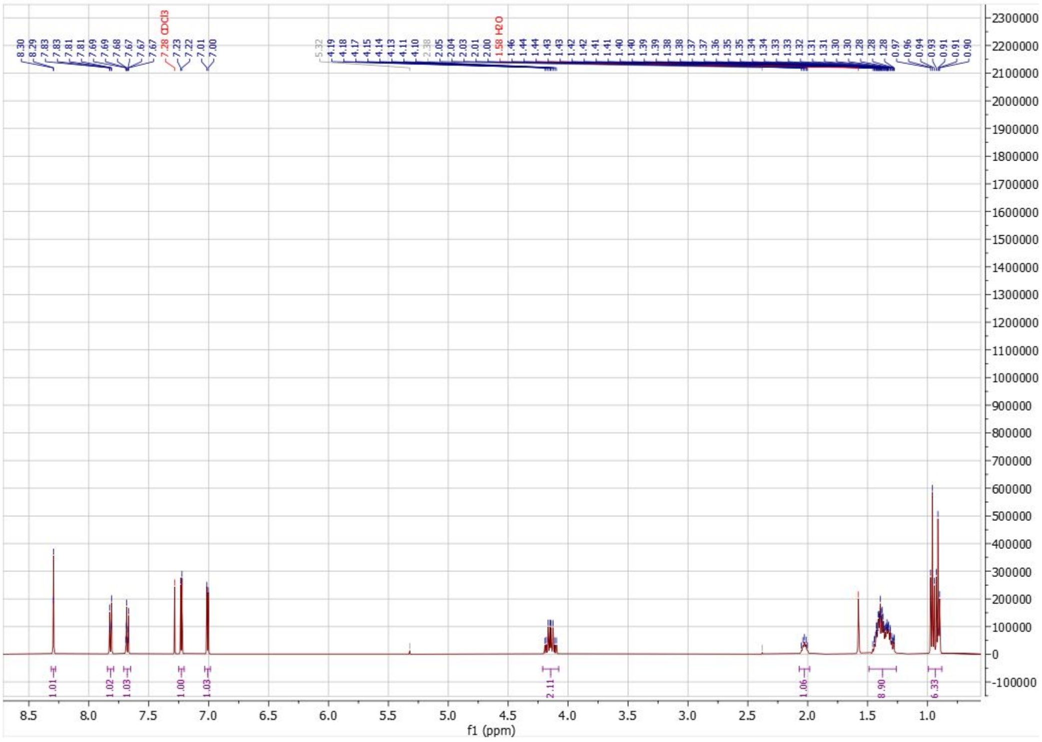

Figure S14. The ^{1}H NMR (400 MHz) spectrum of compound **2** in $CDCl_3$.

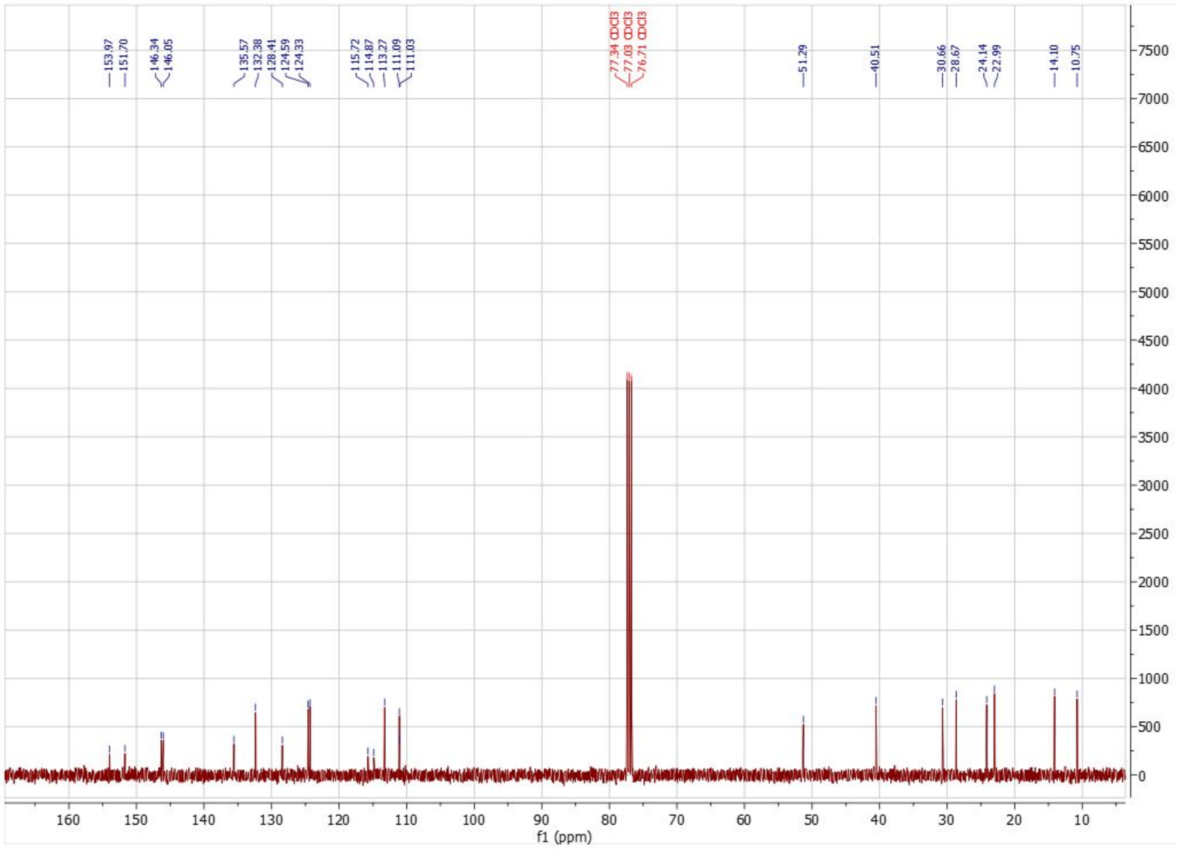

Figure S15. The ^{13}C NMR (101 MHz) spectrum of compound **2** in $CDCl_3$.

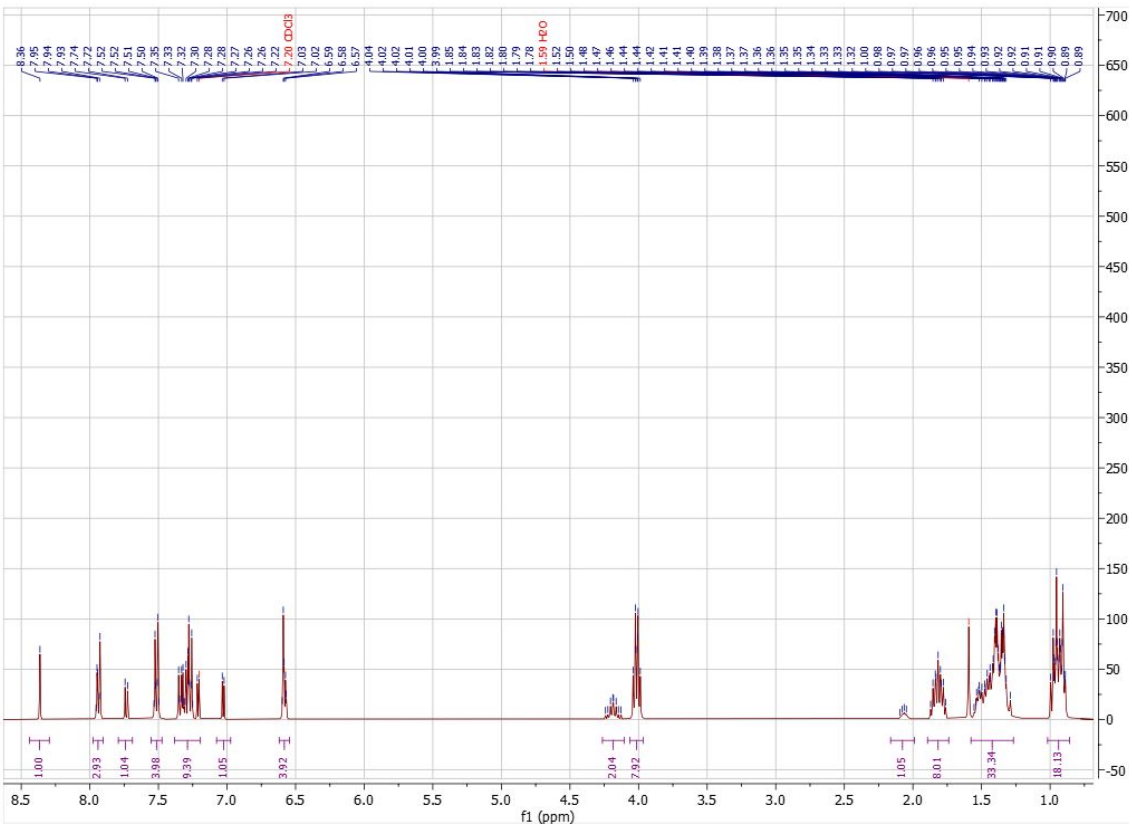

Figure S16. The ^{1}H NMR (400 MHz) spectrum of compound **4** in $CDCl_3$.

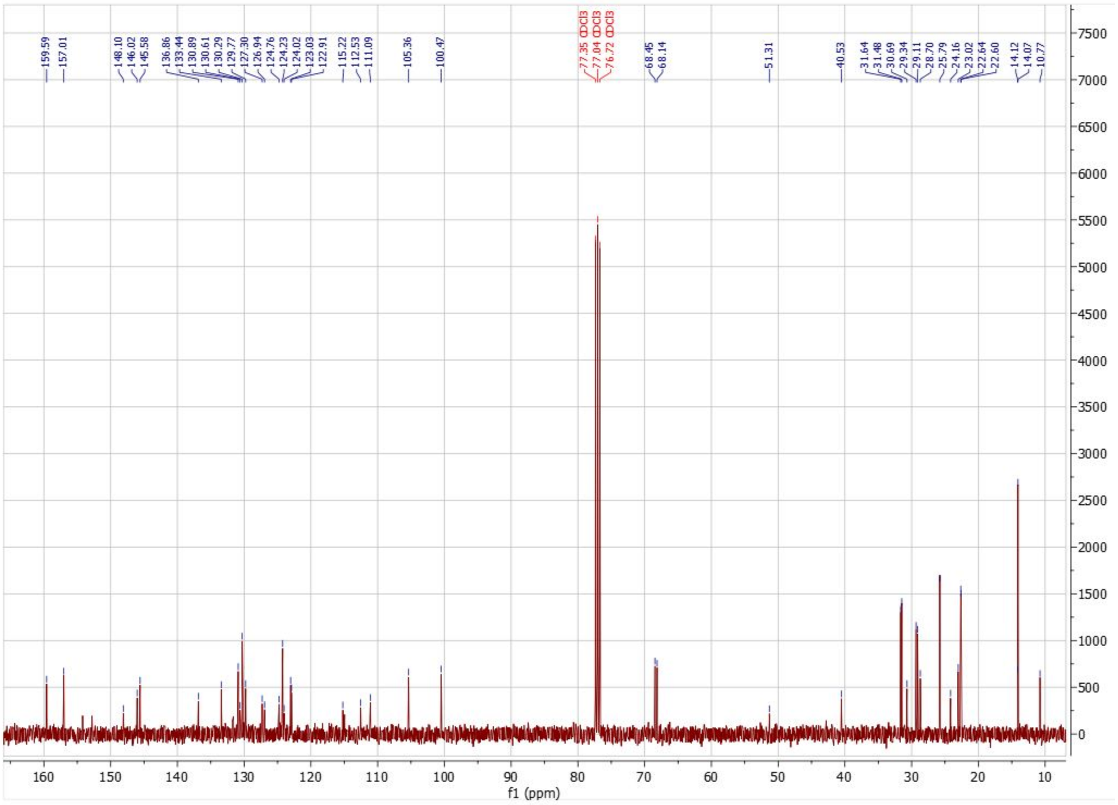

Figure S17. The ^{13}C NMR (101 MHz) spectrum of compound **4** in $CDCl_3$.

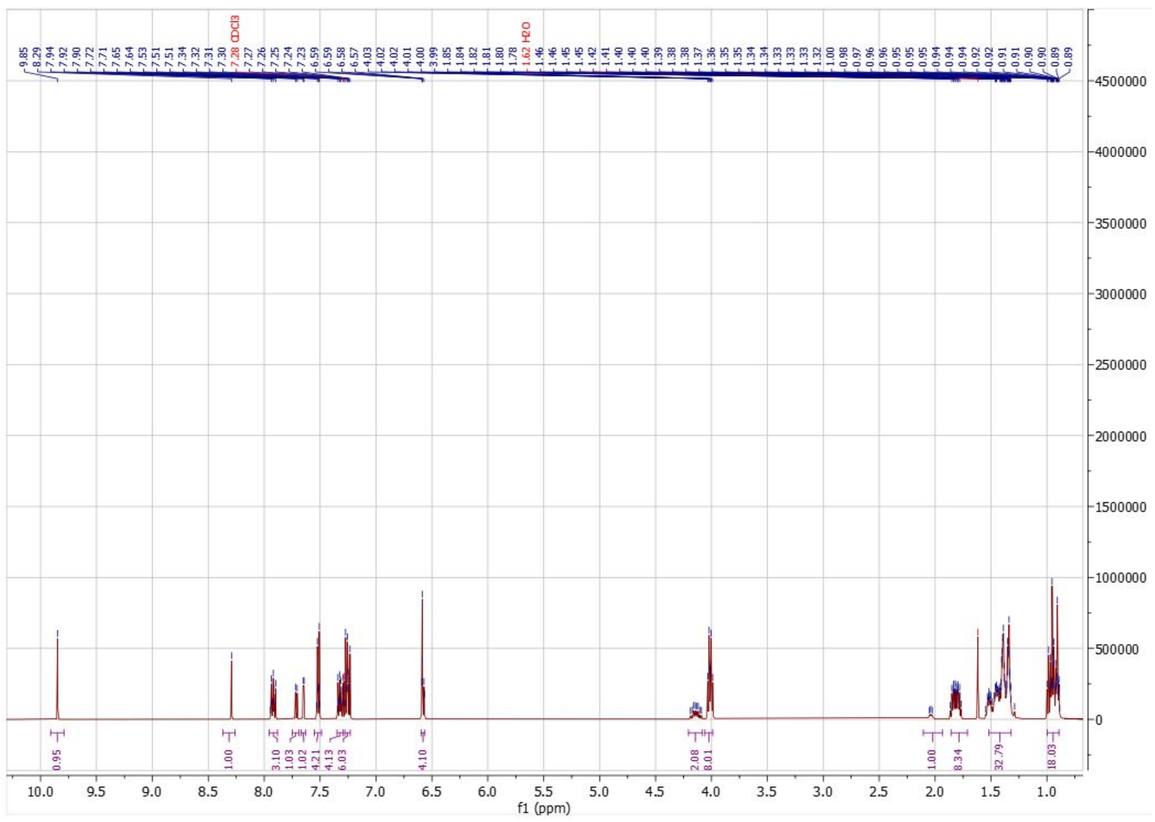

Figure S18. The ^{1}H NMR (400 MHz) spectrum of compound **5** in $CDCl_3$.

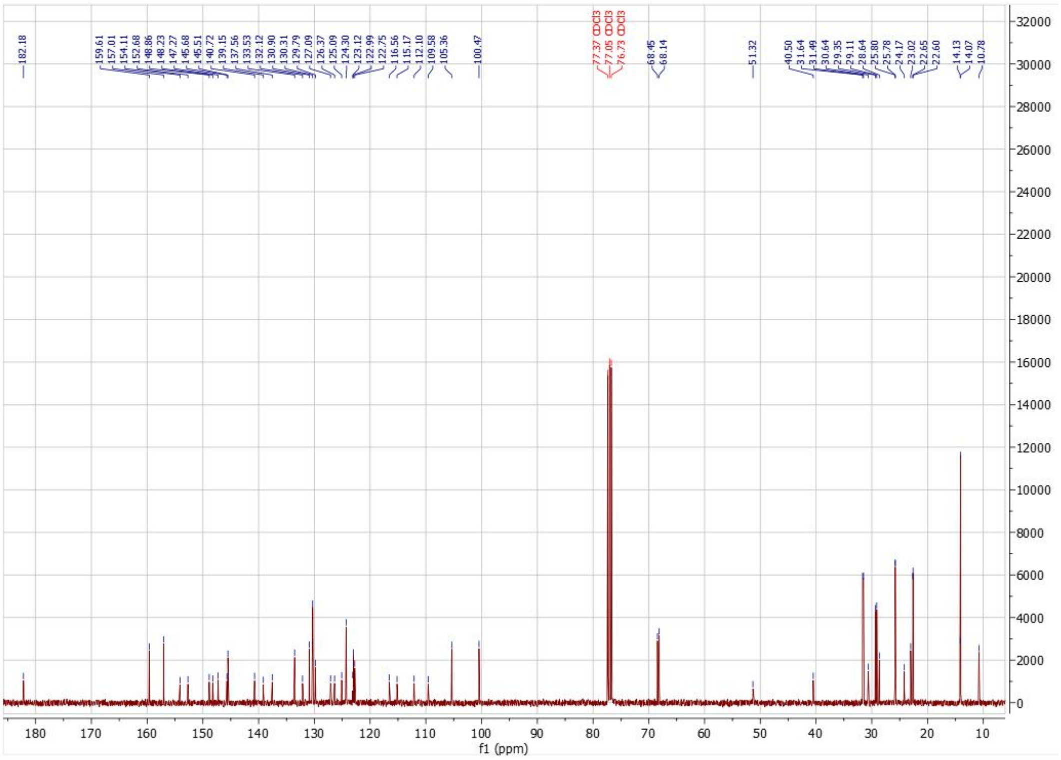

Figure S19. The ^{13}C NMR (101 MHz) spectrum of compound **5** in $CDCl_3$.

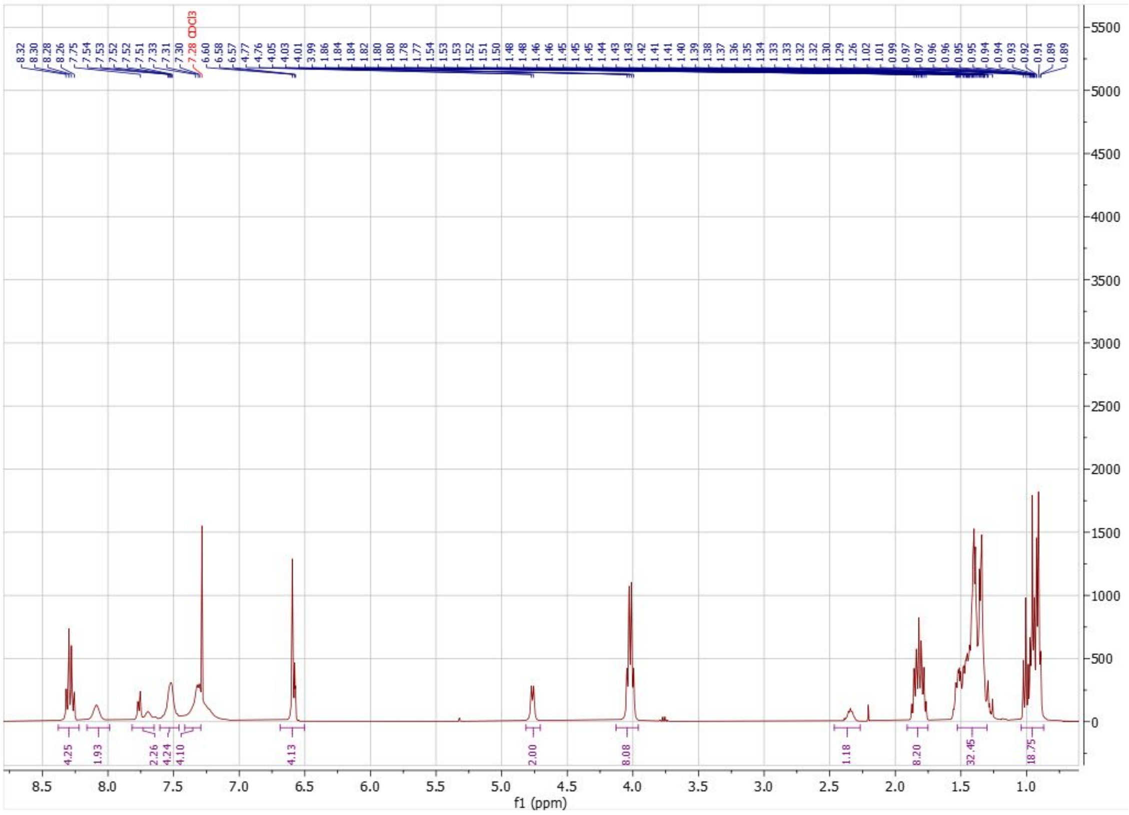

Figure S20. The ^{1}H NMR (400 MHz) spectrum of **H15** in $CDCl_3$.

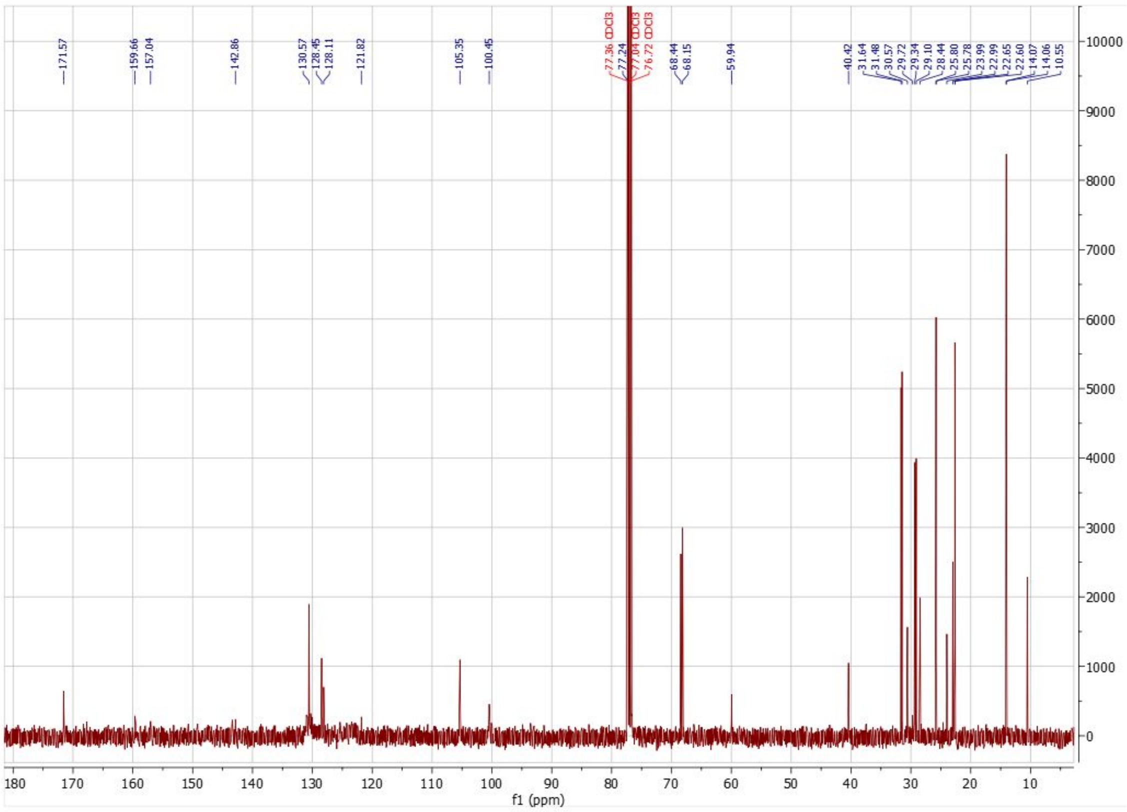

Figure S21. The ^{13}C NMR (101 MHz) spectrum of **H15** in $CDCl_3$.

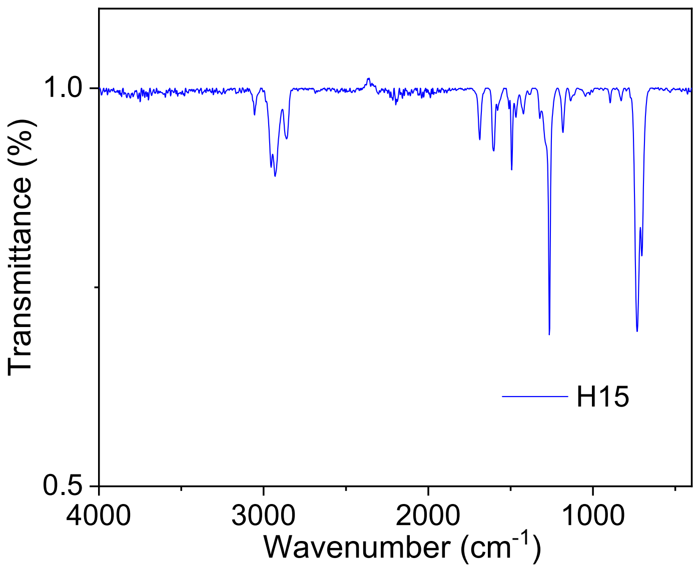

Figure S22. ATR-FTIR spectrum of **H15**.

Mass Spectrum SmartFormula Report

Analysis Info

Analysis Name	D:\Data\MALDI\2025\250804\hwu-H15_0_F23_MS.d	Acquisition Date	8/4/2025 1:34:46 PM
Method	MALDI PepmixII DL.m	Operator	TOF-User
Sample Name	hwu-H15	Instrument / Ser#	timsTOF flex 1893754.30739
Comment	CHCl3, DCTB, MALDI+		

Acquisition Parameter

Source Type	MALDI	Scan Begin	150 m/z
Focus	Not active	Scan End	3300 m/z
		Ion Polarity	Positive

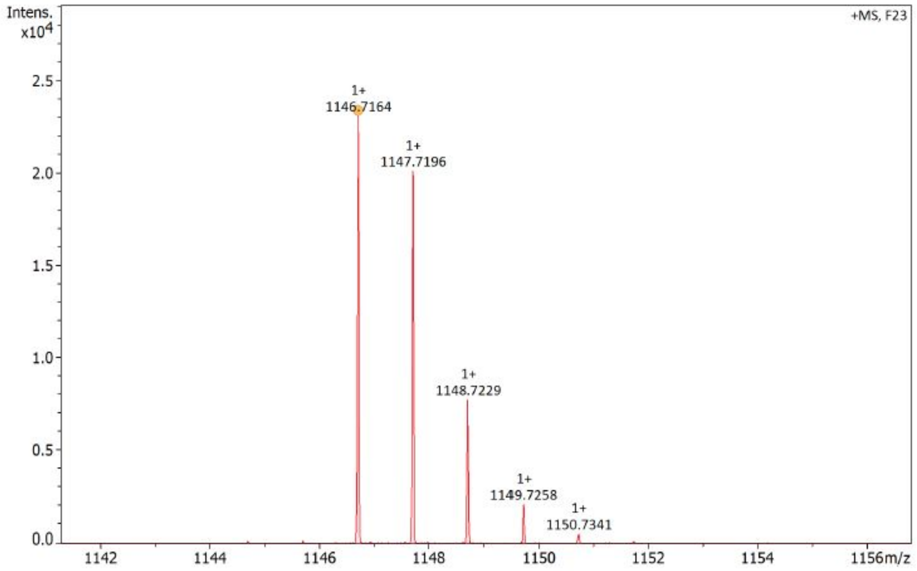

Meas. m/z	Ion Formula	m/z	err [ppm]	err [mDa]	mSigma	e⁻ Conf	z
1146.7164	C75H94N4O6	1146.7168	0.3	0.4	19.9	odd	1+

Figure S23. High resolution mass spectrum of **H15**.

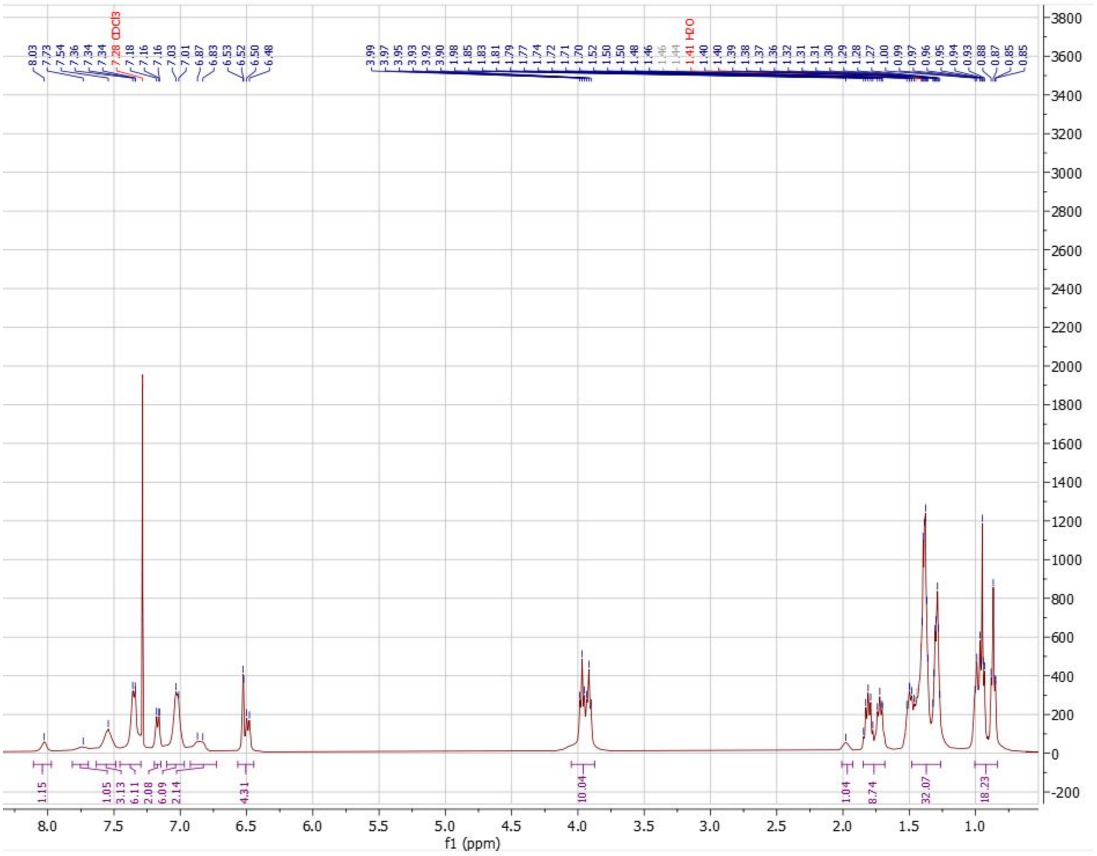

Figure S24. The ^{1}H NMR (400 MHz) spectrum of **H4** in $CDCl_3$.

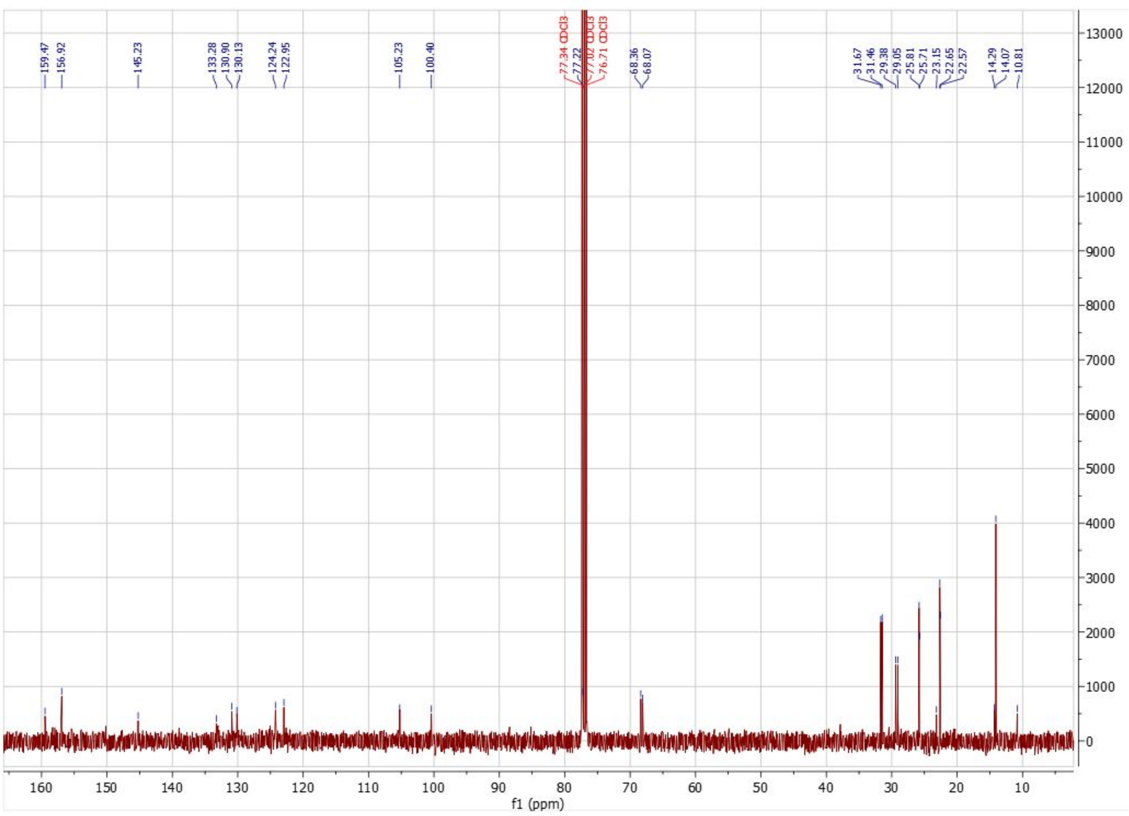

Figure S25. The ^{13}C NMR (101 MHz) spectrum of **H4** in $CDCl_3$.

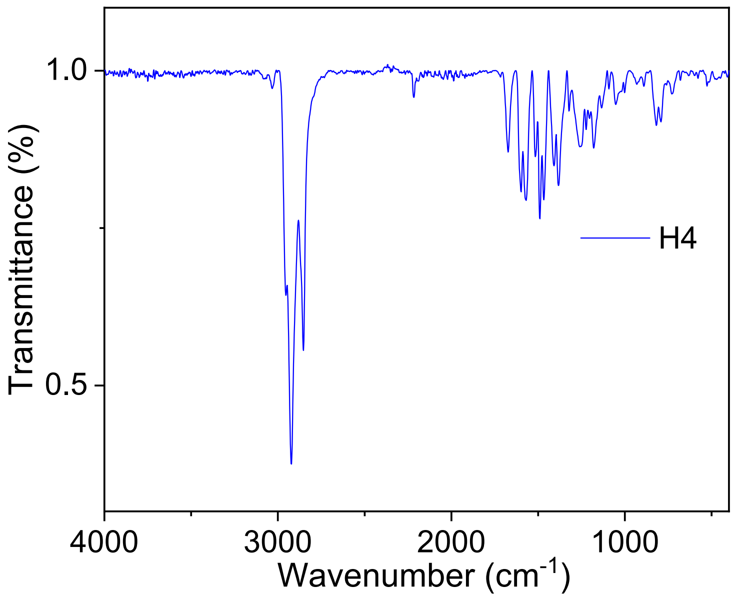

Figure S26. ATR-FTIR spectrum of **H4**.

Mass Spectrum SmartFormula Report

Analysis Info

Analysis Name	D:\Data\MALDI\2025\250311\H4_0_L8_MS.d	Acquisition Date	3/11/2025 3:08:13 PM
Method	MALDI PepmixII DL.m	Operator	TOF-User
Sample Name	H4	Instrument / Ser#	timsTOF flex 1893754.30739
Comment	sample and dctb in CHCl3, MALDI+		

Acquisition Parameter

Source Type	MALDI	Ion Polarity	Positive
Focus	Not active		
Scan Begin	1100 m/z	Set Capillary	3500 V
Scan End	3300 m/z	Set End Plate Offset	-500 V

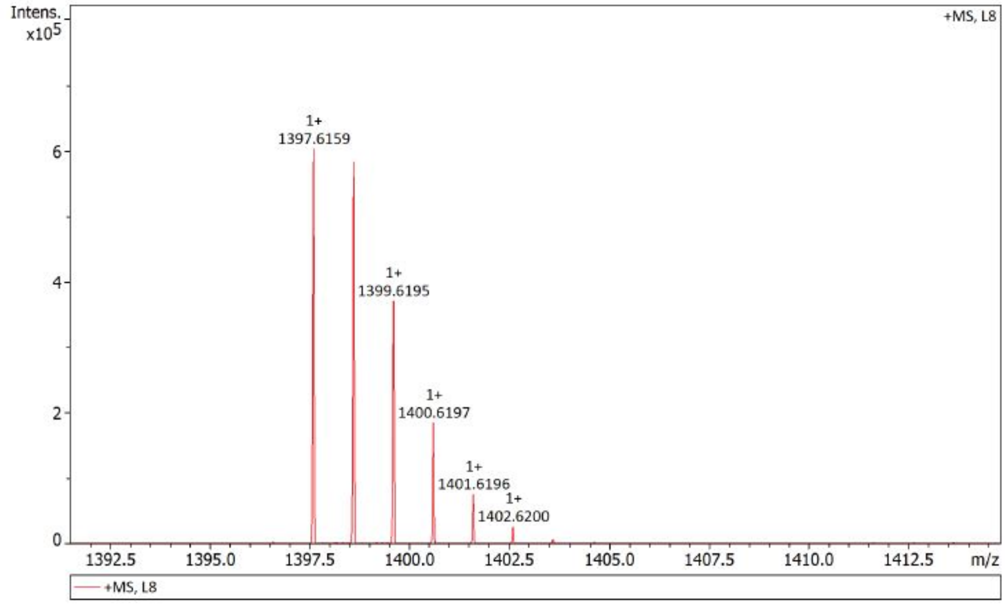

Figure S27. High resolution mass spectrum of **H4**.
